# Clinical effectiveness of gasless laparoscopic surgery for abdominal conditions: systematic review and meta-analysis

**DOI:** 10.1007/s00464-021-08677-7

**Published:** 2021-08-16

**Authors:** N. Aruparayil, W. Bolton, A. Mishra, L. Bains, J. Gnanaraj, R. King, T. Ensor, N. King, D. Jayne, B. Shinkins

**Affiliations:** 1 Leeds Institute of Medical Research at St. James’s, University of Leeds, Leeds, UK; 2 Maulana Azad Medical College, Delhi, India; 3 Karunya University, Coimbatore, India; 4 Nuffield Centre for International Health and Development, Leeds Institute of Health Sciences, University of Leeds, Leeds, UK; 5 Academic Unit of Health Economics, Leeds Institute of Health Sciences, University of Leeds, Leeds, UK; 6 NIHR Global Health Research Group, Surgical Technologies, Clinical Sciences Building, Level 7, Room 7.19, Leeds, LS9 7TF UK

**Keywords:** Gasless laparoscopy, Abdominal wall lift, LMIC, Low resource setting, Rural surgery, Clinical effectiveness, Open surgery, General surgery, Gynaecological surgery

## Abstract

**Background:**

In high-income countries, laparoscopic surgery is the preferred approach for many abdominal conditions. Conventional laparoscopy is a complex intervention that is challenging to adopt and implement in low resource settings. This systematic review and meta-analysis evaluate the clinical effectiveness of gasless laparoscopy compared to conventional laparoscopy with CO_2_ pneumoperitoneum and open surgery for general surgery and gynaecological procedures.

**Methods:**

A search of the MEDLINE, EMBASE, Global Health, AJOL databases and Cochrane Library was performed from inception to January 2021. All randomised (RCTs) and comparative cohort (non-RCTs) studies comparing gasless laparoscopy with open surgery or conventional laparoscopy were included. The primary outcomes were mortality, conversion rates and intraoperative complications. Secondary outcomes: operative times and length of stay. The inverse variance random-effects model was used to synthesise data.

**Results:**

63 studies were included: 41 RCTs and 22 non-RCTs (3,620 patients). No procedure-related deaths were reported in the studies. For gasless vs conventional laparoscopy there was no difference in intraoperative complications for general RR 1.04 [CI 0.45–2.40] or gynaecological surgery RR 0.66 [0.14–3.13]. In the gasless laparoscopy group, the conversion rates for gynaecological surgery were high RR 11.72 [CI 2.26–60.87] when compared to conventional laparoscopy. For gasless vs open surgery, the operative times were longer for gasless surgery in general surgery RCT group MD (mean difference) 10 [CI 0.64, 19.36], but significantly shorter in the gynaecology RCT group MD − 18.74 [CI − 29.23, − 8.26]. For gasless laparoscopy vs open surgery non-RCT, the length of stay was shorter for gasless laparoscopy in general surgery MD − 3.94 [CI − 5.93, − 1.95] and gynaecology MD − 1.75 [CI − 2.64, − 0.86]. Overall GRADE assessment for RCTs and Non-RCTs was very low.

**Conclusion:**

Gasless laparoscopy has advantages for selective general and gynaecological procedures and may have a vital role to play in low resource settings.

**Supplementary Information:**

The online version contains supplementary material available at 10.1007/s00464-021-08677-7.

Globally, general surgical and gynaecological diseases are a significant health burden [1]. Timely access to surgery is cost-effective and can make a substantial contribution to improving global health [2]. The Lancet Commission on Global Surgery has estimated that 143 million additional surgical procedures are needed in Low- and Middle-Income Countries (LMICs) each year to save lives and prevent disability [3].

According to the disease control priorities (DCP-3) on essential surgery, 9% of deaths due to acute abdominal conditions (appendicitis, gallbladder and bile duct disease, hernia, and paralytic ileus/intestinal obstruction) could have been avoided and 6.3% of Disability Adjusted Life Years (DALYs) averted per year if basic surgical care was available in LMICs [1]. The estimate is even higher for maternal and neonatal surgical conditions. Essential surgical procedures are defined as those who have large health burden, cost-effective and can be successfully treated by a surgical intervention [4].

In high-income countries, laparoscopic surgery is associated with better patient outcomes, shorter hospital stays and early return to work when compared to open surgery. These benefits are also recognised in low- and middle-income countries [5], with laparoscopic surgery associated with lower complications, particularly lower surgical site infection rates [6–8].

Although the benefits of laparoscopic surgery are well-recognised, its diffusion in low resource settings of LMICs has been slow. The process of introducing conventional laparoscopic surgery in LMICs is challenging, mainly due to limited infrastructure, resources and lack of training opportunities [9, 10]. In the last decade, due to lower implementation costs, gasless laparoscopy has become increasingly popular for emergency and elective abdominal surgery in low resource settings [4, 11, 12]. In this technique, the surgeon makes a small incision around the umbilicus and inserts a planar or a ring device. This is used to lift the abdominal wall and create the “working space” without the need to insufflate the abdomen with CO_2_ gas. The remaining steps are performed in a similar fashion to conventional laparoscopic surgery.

Recent publications have demonstrated non-inferiority of gasless laparoscopy for general and gynaecological surgery with no difference in peri-operative outcomes when compared to conventional laparoscopy [13, 14]. Non-RCTs comparing gasless to open surgery have also shown favourable outcomes [15, 16]. A Cochrane review of RCTs reported outcomes of gasless as compared to conventional cholecystectomy and another review focussed on the safety of myomectomy [17, 18]. These studies were unable to draw any conclusive evidence of non-inferiority of gasless over conventional or open surgery. To our knowledge, no meta-analysis has been carried out comparing gasless laparoscopy to open surgery or conventional laparoscopy for procedures that include essential general surgical and gynaecological conditions.

## Methods

### Search strategy and selection criteria

**Fig. 1:**
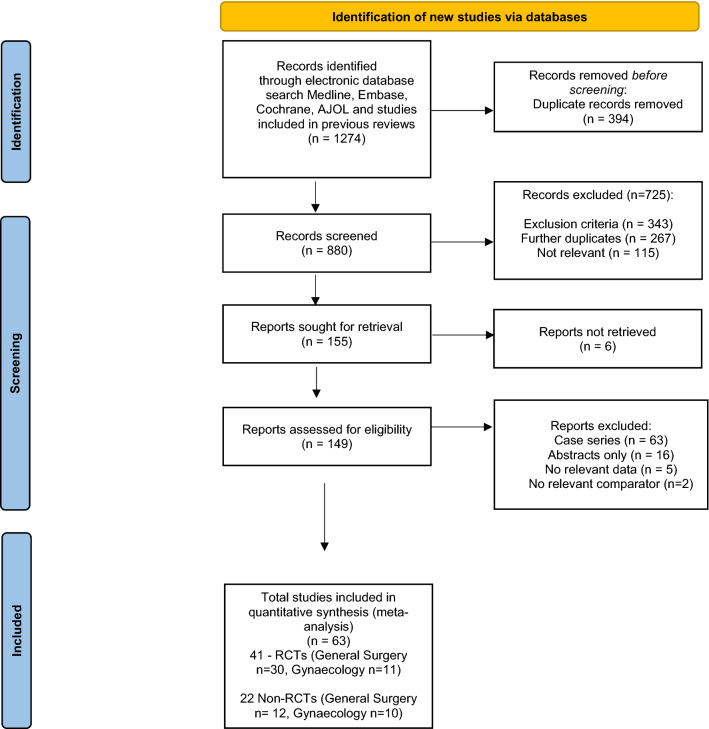
PRISMA flow chart

A systematic review and meta-analysis were conducted and reported following the updated PRISMA guidelines 2020 (Fig. 1) [19].

The bibliographic databases MEDLINE, EMBASE, Global Health, AJOL and Cochrane Library were searched from inception to April 2020 and re-run in January 2021, with no language restrictions, for studies comparing gasless laparoscopy with conventional laparoscopy or open surgery. Additional studies were identified from previously conducted systematic reviews on gasless laparoscopy. Text words and MeSH terms for laparoscopy and minimally invasive surgery were combined with terms related to gasless techniques and excluded non-abdominal procedures using breast, prostat*, urolog*, nephr*, thoracic, endocrine or thyroid. The syntax with search terms is shown in (Supplement search strategies). De-duplication and screening were carried out on EndNote software (X 8.2).

Inclusion of the studies was a stepwise process. Two reviewers (NA and WSB) independently screened the titles and abstracts against the agreed inclusion criteria, and then extracted and selected relevant full-text records. Discrepancies were resolved through discussion at each stage by consensus. Two additional authors (AM & LB) verified the eligibility of inclusion of the studies when necessary.

Included studies compared gasless to conventional laparoscopy, open surgery or both techniques for general surgical and gynaecological conditions in humans. Only RCTs and Non-RCTs were included in the systematic review and meta-analysis. The term non-RCTs used in this review includes comparative prospective and retrospective cohort studies [20]. Case reports, case series or review articles were excluded. The conversion rate for gasless was defined as those cases which were converted to conventional laparoscopy with CO_2_ pneumoperitoneum or open surgery.

### Data analysis

Assessment of both methodological quality and risk of bias was performed by NA and reviewed by WSB independently. Disagreements were resolved by discussion between AM and LB. Included studies were stored on an Excel spreadsheet for data extraction. Details extracted included: study design, follow-up period, device, operated organ, comparators and sample size. The primary outcomes were mortality, conversion rate and intraoperative complications. Secondary outcomes were overall complications (inclusive of intraoperative complications), operative time and length of operative stay.

Risk‐of‐bias assessment for each outcome in randomised controlled trials (RCTs) was done using the risk of bias—2 (RoB-2) tool for six domains: randomisation process, deviation from the intended interventions, missing outcome data, measurement of the outcome, selection of the reported result and overall risk of bias [21]. The risk of bias for each outcome in the non-RCT studies was assessed with ROBINS-I tool with seven domains: confounding, selection of participants, classification of interventions, deviations from intended interventions, missing data, measurement of outcomes and selection of the reported result and overall [22].

Domain level and overall risk of bias judgements for the outcomes in RCTs were assessed as having either a low, some concerns or high risk of bias according to the RoB-2 tool. For ROBINS—I tool, results of non-RCT studies were assessed for domain level and overall risk of bias as having either low risk, moderate, severe, critical and no information. Within each study, we summarised the risk of bias for individual outcome of interest for each comparator using RoB-2 and ROBINS-I tool. Effect measures for outcomes which were ‘not estimable’ were excluded from risk of bias assessment. In addition, we used the GRADE assessment of the quality of evidence to summarise the primary and secondary outcomes in RCTs and non-RCT studies [23]. Within GRADE, certainty assessment for RCTs was based on the following parameters: risk of bias, Inconsistency, Indirectness and Imprecision. A summary of evidence table was compiled using GRADEpro GDT software (McMaster University, 2020) for general surgery and gynaecology RCT and non-RCT studies. The interpretation of the quality of evidence for RCT and non-RCT studies was done independently based on the effect measure and not compared to each other.

In the meta-analysis, the effect measures were estimated for each comparator using the Forest Plot. To address heterogeneity, we performed subgroup analyses for RCT and Non-RCT studies for general surgery and gynaecology procedures, for each outcome of interest. Due to assumed heterogeneity of the data, inverse variance random-effects models were used for dichotomous and continuous data, with significance set at 95% confidence interval or p-value of < 0.05 as significant. We evaluated heterogeneity using *I*^*2*^ statistics for between study variance for the subgroup analysis and pooled effect. For conversion rates, we used a funnel plot to assess for publication bias in the included studies.

The overall treatment effect was calculated as a weighted average of events for individual summary statistics. Measures of effect: for dichotomous data—risk ratio (RR) with a 95% confidence interval (CI) was used. Continuous data were analysed using mean difference (MD) with 95% CI. Subgroup analysis was also performed for studies included in the LMIC population groups.

Analyses were performed using Review Manager software (RevMan version 5.4; The Nordic Cochrane Centre, Copenhagen, Denmark) [24]. The study was prospectively registered on PROSPERO CRD42020173264 https://www.crd.york.ac.uk/prospero/display_record.php?RecordID=173264.

### Role of the funding source

The funder of the study had no role in study design, data collection, data analysis, data interpretation or writing of the report. The corresponding author had full access to all the data in the study and had final responsibility for the decision to submit for publication.

## Results

We identified 1274 records on initial screening (Fig. 1). After removing duplicates, 880 records were screened using abstracts. At this stage, 731 studies that did not meet the inclusion criteria were excluded. Out of 155 reports sought for retrieval, 6 reports were not retrieved. Based on a full-text review of the remaining 149 studies, a further 86 studies were excluded leaving a total of 63 studies (41 RCTs and 22 non-RCT).

A total of 3,648 patients were included in the systematic review and meta-analysis. The RCTs of general surgical (*n* = 30) and gynaecological procedures (*n* = 11) consisted of 2263 patients (Table 1 supplement) [13, 25–64]. The majority of the general surgical procedures included in the review were cholecystectomy, appendicectomy and diagnostic laparoscopy. In the gynaecology group; adnexal procedures, myomectomies and hysterectomies were included. A detailed breakdown can be found in Supplement Tables 1 and 2.

A detailed summary of the risk of bias for RCTs using RoB—2 tool is listed in Table 3 Supplement. In the non-RCT group, there were 12 general surgery and 10 gynaecology studies, with a total of 1,385 patients (Table 2 Supplement) [14–16, 65–83]. The overall result level assessment for each outcome varied between low to high risk of bias. Complication rates were assessed at having ‘some concerns’ to ‘high risk’ of bias due to issues around randomisation process or measurement and selection of outcome domains. Conversion rates, operative time and LoS were assessed as ‘low’ to ‘some concerns’. The summary of the risk of bias of non-RCTs according to the ROBINS-I tool is listed in Table 4 Supplement. Overall risk of bias for complication and conversation rates were assessed as ‘moderate’ to ‘serious’ due to confounding, selection of participants, measurement and selection of results domain. Overall risk of bias for operative times and LoS were reported at low to moderate.

A detailed level of evidence is summarised in GRADE assessment tables for RCTs and non-RCTs (Tables 5–8) comparing gasless to conventional laparoscopy and open surgical technique. It provides relative and absolute effect measures of the results summarised above and gives certainty of evidence for each comparator in each domain. Most studies are graded as low or very low level of evidence.

### Primary outcomes

#### Mortality

Forty six percent (19/41) of the RCTs reported no mortality. Only 29% (12/41) of the studies reported short-term follow-up timings, apart from one study which followed up patients for a year [25]. Remaining studies did not report the duration of follow-up of the enrolled patients. In non-RCT studies, 27% reported no mortality, except one death which was reported due to malignancy 45 days post-operatively [16].

### Intraoperative complications

#### Gasless vs conventional laparoscopy

In the gasless laparoscopy group, procedure-specific complications were reported as small bile leak [26], bile duct stricture [27], bleeding & bruising [25, 27–30] and perforation—small bowel and uterus [27, 30]. Similar complications were reported in those who had conventional laparoscopy. For intraoperative (procedure-specific) complications in the general surgery (19 studies, 806 patients) and gynaecology (6 studies, 636 patients). RCTs subgroup analysis, there was no statistically significant overall difference between the gasless and conventional surgery groups RR 1.04 [CI 0.45, 2.40] and RR 0.66 [CI 0.14, 3.13] respectively (Fig. 2 Supplement). No difference was found in the gynaecology non-RCT subgroup (4 studies, 336 patients).

#### Gasless laparoscopy vs open surgery

One study comparing gasless versus open surgery versus conventional laparoscopy (CO_2_ insufflation) for cholecystectomy had hepatic bleeding in all arms except in the open technique. The intraoperative risk of complication for this study (10 patients) when comparing gasless vs open was high, but statistically not significant RR 3.0 [CI 0.15, 59.89] [28]. The intraoperative risk for gasless versus open gynaecological procedures (2 studies, 180 patients) was not estimable due to zero events in both groups.

### Secondary outcomes

#### Conversion rates

No difference was observed in the conversion rate for general surgical procedures in RCTs (12 studies, 713 patients) for gasless when compared to conventional laparoscopy RR 1.49 [CI 0.71, 3.14], *I*^2^ 11% (Fig. 3 Supplement). In the RCT subgroup analysis, the conversion rate was significantly higher for gynaecological procedures (3 studies, 534 patients) for gasless when compared to conventional laparoscopy RR 11.72 [CI 2.26, 60.87], *I*^2^ 0%). There was no difference in the conversion rate in non-RCT studies: general surgery (4 studies, 328 patients) RR 0.86 [CI 0.41, 1.83], *I*^2^ 0% and gynaecology (7 studies, 569 patients) RR 0.90 [CI 0.15, 5.21], *I*^2^ 0%. The funnel plot (Fig. 4 Supplement) did not indicate publication bias with an even distribution of the included studies to estimate the effect of conversion rate.

### Overall complications

#### Gasless vs conventional laparoscopy

There was no difference in the overall complication rate in the gasless group when compared to conventional laparoscopy in the general RCT subgroup (19 studies, 829 patients) RR 0.89 [CI 0.56, 1.43] *I*^2^ 17% (Fig. 5 Supplement). No difference was found in the gynaecology RCT subgroup (6 studies, 638 patients) RR 1.08 [CI 0.39, 2.99], *I*^2^ 0%. The results of the non-RCT subgroup analyses did not show any difference in the overall complication rates, general surgery (4 studies, 316 patients) RR 0.62 [CI 0.24, 1.64] and gynaecology (2 studies, 230 patients) RR 0.23 [CI 0.01, 4.28].

#### Gasless laparoscopy vs open surgery

No difference was noted in the subgroup analysis of the overall complications when comparing gasless versus open surgery in the general surgery RCT group (1 study, 10 patients) RR 1.0 [CI 0.08, 11.93]. (Fig. 6 Supplement). Results of the gynaecology RCT subgroup were not estimable due to zero events in either group (2 studies, 180 patients). Similarly, no statistical difference was seen in the overall complication rates in gasless versus open technique in the general surgical non-RCT (4 studies, 186 patients) RR 0.84 [CI 0.34, 2.06], *I*^2^ 0% and gynaecology non-RCT (3 studies, 227 patients) RR 0.82; [CI 0.41, 1.66], *I*^2^ 0%. There was no evidence of heterogeneity for the results of the non-RCTs.

### Operative times

#### Gasless vs conventional laparoscopy

Operative times were higher for gasless general surgery RCTs (25 studies, 1046 patients) when compared to conventional laparoscopy MD 8.53 [CI 4.68, 12.38], *I*^2^ 64% (Fig. 7 Supplement). No difference was seen in the operative times for gynaecology RCTs (6 studies, 296 patients) MD − 0.02 [CI − 8.90, 8.86], *I*^2^ 0%. There were no statistically significant differences in the operative times in the general surgery (5 studies, 355 patients) MD 0.52 [CI − 4.87, 5.92], *I*^2^ 47% and gynaecology non-RCT (6 studies, 566 patients) MD 8.16 [CI − 1.87, 18.19], *I*^2^ 86% non-RCT subgroup analyses.

#### Gasless laparoscopy vs open surgery

One study (10 patients) included in the general surgery RCT group showed longer operative time for gasless when compared to open surgery MD 10 [CI 0.64, 19.36] (Fig. 8 Supplement). In the gynaecology RCTs (2 studies, 180 patients), operative times were significantly shorter in the gasless group when compared to open technique MD − 18.74 [CI − 29.23, − 8.26], *I*^2^ 55%. The operative times in the gynaecology non-RCT (3 studies, 227 patients) were longer for gasless when compared to open surgery, but results were not statistically significant MD 25.11 [CI − 3.34, 53.55] *I*^2^ 94%. No difference was found in those who had general surgical procedures (8 studies, 330) in the open versus gasless non-RCT subgroup MD 3.83 [CI − 22.52, 30.18], *I*^2^ 95%.

### Length of stay

#### Gasless vs conventional laparoscopy

In the subgroup analysis, no difference was found in the length of hospital stay for general surgical RCTs (10 studies, 452 patients) for gasless when compared to conventional laparoscopy MD 0.24 [CI − 0.14, 0.62], I^2^ 57% (Fig. 9 Supplement). A statistically significant shorter length of stay was noted in those who had gasless surgery in the gynaecology RCT (3 studies, 493 patients) MD − 0.93 [CI − 1.27, − 0.58], *I*^2^ 24% but not in the gynaecology non-RCT group (4 studies, 263 patients) MD − 1.10 [CI − 0.22, 0.02], *I*^2^ 0% when compared to conventional laparoscopic surgery. No difference was seen in the general surgery non-RCT group (3 studies, 204 patients) MD 0.04 [CI − 0.80, 0.88], *I*^2^ 0%.

#### Gasless laparoscopy vs open surgery

In the gasless versus open subgroup analysis, shorter length of stay was observed in those who had gasless compared to open technique in the general surgery non-RCT (8 studies, 330 patients) MD − 3.94 [CI − 5.93, − 1.95], *I*^2^ 95% and gynaecology non-RCT (3 studies, 227 patients) MD − 1.75 [CI − 2.64, − 0.86], *I*^2^ 91%. There was no difference in the general surgery RCT subgroup (2 studies, 110 patients) MD − 2.46 [CI − 5.23, 0.30], *I*^2^ 72% (Fig. 10 Supplement).

#### LMIC subgroup analyses

Due to the paucity of data from LMICs, only two general surgery RCTs from India [13, 31] and two non-RCTs from Ukraine [65, 66] comparing gasless versus conventional laparoscopy were included in a subgroup analysis (Figs. 11–13 supplement). For RCTs (140 patients), there was no difference in the operative times MD 4.82 [CI − 7.12, 16.77], conversion rates RR 1.67 [CI 0.42, 6.60] or overall complication rates RR 1.39 [CI 0.69, 2.79]. For non-RCTs (151 patients), there was no statistically significant differences in operative times MD 0.97; [CI − 2.08, 4.03], conversion rates RR 0.74 [CI 0.30, 1.84] and overall complications rates RR 0.39 [CI 0.15, 1.05].

## Discussion

This is the largest systematic review and meta-analysis on gasless laparoscopic surgery for general and gynaecological conditions to date. Most systematic reviews and meta-analysis in the past focussed on safety of cholecystectomy or myomectomies. This review primarily evaluates procedures that could be amenable for gasless laparoscopic intervention over open surgery for a low resource setting.

Due to the weaker level of evidence of several underpowered RCTs and considerable heterogeneity of non-RCTs, it was not possible to draw firm conclusions about the non-inferiority of gasless over conventional laparoscopy or open surgery. Several studies included in the review had incomplete reporting of the methodology, which increased the risk of bias in how RCTs and non-RCTs were assessed. Including zero events in both arms in all the analyses was considered as a robust way of communicating and reporting the results in the meta-analysis [84].

Nearly half of the included RCTs reported mortality and only one third of those reported on short-term follow-up time. Although the majority of studies included in the review were from HICs, the reporting of mortality and follow-up data were better in LMIC studies. High quality randomised intervention studies in low resource settings with a longer follow-up period may not be a feasible starting point to evaluate an intervention. Limited research infrastructure and under funded health systems already bring a lot of pressure on health institutions based in the LMICs. For low resource settings, implementation research methodology such as process evaluation and cohort studies are robust strategies in evaluating complex interventions like laparoscopic surgery in evaluating longer term implications on morbidity and mortality [85, 86].

Conversion rates were considerably higher for gasless compared to conventional laparoscopy in the gynaecology RCT group, but no statistical differences were found in general surgery or the non-RCT subgroup analyses. A further breakdown of this analysis shows that, in 1990s, with the peak interest in gasless technique, studies reported higher conversion rates for adnexal procedures with smaller sample sizes [32, 33]. A myomectomy study by Wang and colleagues in 2011, with a larger sample size, did not report any conversions in either group [34]. One explanation given by Cravello and colleagues was the location of adnexal organs in obese patients resulting in limited views of the pelvis when using the gasless technique [32]. Extreme Trendelenburg position did not improve the view and led to conversion to conventional laparoscopy.

Overall, the reasons reported for conversion from gasless to either CO_2_ pneumoperitoneum or open were (1) limited exposure, (2) adhesions, (3) intestinal loops, (4) lower pelvic organs, (5) higher BMIs and (6) limited visualisation of lateral aspects of the abdomen (dependent on the type of abdominal lift device used). Type of pelvic surgery, operator experience, patient body habitus and learning curve may also contribute to the higher conversion rates.

The included studies generally attributed less attention on reporting the training of surgeon and its impact on the outcomes. Several barriers to laparoscopic training already exist in low resource setting, and focussed training in gasless laparoscopy could simplify adoption of laparoscopy over open surgery [5, 10, 87, 88]. In the absence of CO_2_ pneumoperitoneum, several reviews and recently conducted RCT by Mishra and colleagues highlighted fewer peri-operative complications and better physiological tolerance with gasless technique [13, 17, 18, 89]. Wang and colleagues discuss that during gasless laparoscopy, instruments traditionally used for open gynaecological procedures simplify intraoperative suturing of the uterus, increase precision during ligation and reduce complications [34].

Longer operative times could be attributed to the procedures’ complexities within each subgroup or limited views whilst operating. Hence, device modification is essential to provide uniform lift of the abdominal wall, allowing better visualisation of the peritoneal cavity and achieving comparable views to conventional laparoscopy. The operative times were considerably shorter for gynaecology RCTs but longer for non-RCTs when comparing gasless versus open. The gynaecology RCTs included the procedure of myomectomy, whereas the non-RCTs had hysterectomies and adnexal surgeries. Longer operative times could be attributed to the complexity of the procedures or limited views due as discussed earlier.

The length of hospital stay (LoS) was shorter in patients who underwent less complicated gynaecological procedures using the gasless technique when compared to conventional laparoscopy. The LoS was also considerably shorter for those who underwent gasless when compared to the open surgical technique. A systematic review for gasless myomectomy conducted by Liu and colleagues also found shorter length of stay [18].

Several studies included in the analysis comparing gasless versus open surgery had complex abdominal procedures that may not be suitable for surgeons with limited experience. Major gastrointestinal and gynaecological resectional surgery is not suitable for the gasless approach or for undertaking in level 1 district hospitals, which lack the necessary perioperative support. Such major surgery demands a high level of expertise and sub-specialisation which is concentrated in level 3 facilities. Rather, gasless laparoscopy is best suited to diagnostic procedures or simple, single-quadrant resection, such as appendicectomy, cholecystectomy, salpingectomy, tubal ligation, oophorectomy and myomectomy and in non-obese patients with lower anaesthetic risk.

The introduction of laparoscopic surgery in resource limited settings faces several challenges due to the lack of infrastructure, training opportunities, surgical hierarchy, limited workforce and financial constraints [9, 90]. Nearly 90% of gastrointestinal procedures in low-income settings are operated using open surgery and have higher complication rates compared to laparoscopy [6–8]. The findings of this study should encourage policymakers in low resource settings to prioritise minimal access surgery over open surgery in selective general and gynaecological procedures. Gasless laparoscopy has the potential to be a cost-effective technology to accelerate the adoption of minimally invasive surgery in low resource settings.

There were several limitations to this review. Most studies did not grade the complications resulting from the surgical procedure, which restricts the measurement of the quality of improving health care delivery [91]. The severity of intra- and postoperative complications may vary: a comparison of overall complications may miss unequally distributed severe complications between interventions. Therefore, these findings should be interpreted with great caution due to this limitation.

No subgroup analysis was conducted based on the surgical expertise, type of anaesthesia, ASA score or the use of different abdominal wall lift devices as majority cases were done under general anaesthesia in low-risk patients. Previous operative experience, hours of training, type of lift device and pain scores were either under-reported or disparate across studies to allow meaningful sub-group analyses. Some analyses increased heterogeneity due to the inclusion of different types of procedures within a study. Most studies conducted in HICs and tertiary hospitals of LMICs are less relevant in a low resource setting and are less likely to give a plausible explanation for the outcomes expected. Hence, this review’s findings cannot be generalised and should act as a guide to conduct high quality studies relevant to the context and address the burden of surgical diseases due to abdominal conditions.

## Author Contributions

NA, DGJ, BS, TE and RK came up with the research question and designed the protocol. NA did the literature search, study selection, quality assessment and data extraction. WB independently reviewed title, abstract, full text review and quality of selected studies. The selected studies were reviewed by AM and LB. JG expert in gasless provided critical comments. NK assisted with the search strategies. NA did the statistical analysis with advice from BS. NA wrote the manuscript, designed the tables, figures and appendices. DGJ, BS, TE and RK contributed to critical revisions. All authors approved final manuscript.

## Supplementary Information

Below is the link to the electronic supplementary material.

Supplementary file1 (DOCX 163 kb)

Supplementary file2 (DOCX 4129 kb)

Supplementary file3 (DOCX 16 kb)

## References

[1] Bickler SW, Weiser TG, Kassebaum N, Higashi H, Chang DC, Barendregt JJ, Noormahomed EV, Vos T (2015). Global burden of surgical conditions. Dis Control Priorities Essent Surg.

[2] Mock C, Cherian M, Juillard C, Donkor P, Bickler S, Jamison D, McQueen K (2010). Developing priorities for addressing surgical conditions globally: furthering the link between surgery and public health policy. World J Surg.

[3] Meara JG, Leather AJ, Hagander L, Alkire BC, Alonso N, Ameh EA, Bickler SW, Conteh L, Dare AJ, Davies J, Dérivois Mérisier E, El-Halabi S, Farmer PE, Gawande A, Gillies R, Greenberg SL, Grimes CE, Gruen RL, Adan Ismail E, Buya Kamara T, Lavy C, Lundeg G, Mkandawire NC, Raykar NP, Riesel JN, Rodas E, Rose J, Roy N, Shrime MG, Sullivan R, Verguet S, Watters D, Weiser TG, Wilson IH, Yamey G, Yip W, Greenberg SL, Raykar NP, Riesel JN, Kong Chian L, eld N, Leather AJM, Hagander L, Alkire BC, Alonso N, Ameh EA, Bickler SW, Conteh L, Dare AJ, Davies J, Mérisier ED, El-Halabi S, Farmer PE, Gawande A, Gillies R, Greenberg SLM, Grimes CE, Gruen RL, Ismail EA, Kamara TB, Lavy C, Lundeg G, Mkandawire NC, Raykar NP, Riesel JN, Rodas E, Rose J, Roy N, Shrime MG, Sullivan R, Verguet S, Watters D, Weiser TG, Wilson IH, Yamey G, Yip W, Dérivois Mérisier E, El-Halabi S, Farmer PE, Gawande A, Gillies R, Greenberg SLM, Grimes CE, Gruen RL, Ismail EA, Kamara B, Lavy C, Lundeg G, Mkandawire NC, Raykar NP, Riesel JN, Rodas E, Rose J, Roy N, Shrime MG, Sullivan R, Verguet S, Watters D, Weiser TG, Wilson IH, Yamey G, Yip W, Greenberg SLM, Riesel JN, Lundeg, G., Mkandawire NC, Raykar NP, Riesel JN, Rodas E, Rose J, Roy N, Shrime MG, Sullivan R, Verguet S, Watters D, Weiser TG, Wilson IH, Yamey G, Yip W (2015) The lancet commissions global surgery 2030: evidence and solutions for achieving health, welfare, and economic development. Lancet Comm 386: 569.

[4] Mock C, Donkor P, Gawande A, Jamison D, Kruk M, Debas H (2015) Essential surgery. Disease Control Priorities, 3rd edn. 10.1596/978-1-4648-0346-8_ch17 26740991

[5] Chao TE, Mandigo M, Opoku-Anane J, Maine R (2016). Systematic review of laparoscopic surgery in low- and middle-income countries: benefits, challenges, and strategies. Surg Endosc.

[6] Collaborative GlobSurg (2018). Laparoscopy in management of appendicitis in high-, middle-, and low-income countries: a multicenter, prospective, cohort study. Surg Endosc.

[7] Collaborative GlobSurg (2018). Surgical site infection after gastrointestinal surgery in high-income, middle-income, and low-income countries: a prospective, international, multicentre cohort study. Lancet Infect Dis.

[8] Danwang C, Bigna JJ, Tochie JN, Mbonda A, Mbanga CM, Nzalie RNT, Guifo ML, Essomba A (2020). Global incidence of surgical site infection after appendectomy: a systematic review and meta-analysis. BMJ Open.

[9] Choy I, Kitto S, Adu-Aryee N, Okrainec A (2013). Barriers to the uptake of laparoscopic surgery in a lower-middle-income country. Surg Endosc.

[10] Wilkinson E, Aruparayil N, Gnanaraj J, Mishra A, Bains L, Bolton W, Brown J, Jayne D (2020). Barriers and facilitators of laparoscopic surgical training in rural north-east India: a qualitative study. Int J Surg Glob Heal.

[11] Gnanaraj J, Rhodes MJG (2016). Laparoscopic surgery in middle- and low-income countries: gasless lift laparoscopic surgery. Surg Endosc.

[12] Bolton WS, Aruparayil N, Quyn A, Scott J, Wood A, Bundu I, Gnanaraj J, Brown JM, Jayne DG (2019). Disseminating technology in global surgery. Br J Surg.

[13] Mishra A, Bains L, Jesudin G, Aruparayil N, Singh R, Shashi (2020). Evaluation of gasless laparoscopy as a tool for minimal access surgery in low-to middle-income countries: a phase II noninferiority randomized controlled study. J Am Coll Surg.

[14] Kim MK, Hwang JH, Kim J-H, Kim SR, Lee SB, Kim BW (2020). Gasless total laparoscopic hysterectomy with new abdominal-wall retraction system. JSLS J Soc Laparoendosc Surg.

[15] Akira S, Yamanaka A, Ishihara T, Takeshita T, Araki T (1999). Gasless laparoscopic ovarian cystectomy during pregnancy: comparison with laparotomy. Am J Obstet Gynecol.

[16] Huang CC, Yang CY, Wu MH, Wang MY, Yeh CC, Lai IR, Chen CN, Lin MT (2010). Gasless laparoscopy-assisted versus open resection of small bowel lesions. J Laparoendosc Adv Surg Tech.

[17] Gurusamy KS, Koti R, Davidson BR (2013). Abdominal lift for laparoscopic cholecystectomy. Cochrane Database Syst Rev.

[18] Liu QW, Han T, Yang M, Tong XW, Wang JJ (2016). A systematic review on efficacy and safety of gasless laparoscopy in the management of uterine leiomyoma. J Huazhong Univ Sci Technol.

[19] Page MJ, Mckenzie JE, Bossuyt PM, Boutron I, Hoffmann TC, Mulrow CD, Shamseer L, Tetzlaff JM, Akl EA, Brennan SE, Chou R, Glanville J, Grimshaw JM, Hróbjartsson A, Lalu MM, Li T, Loder EW, Mayo-Wilson E, Mcdonald S, Mcguinness LA, Stewart LA, Thomas J, Tricco AC, Welch VA, Whiting P, Moher D (2021) The PRISMA 2020 statement: an updated guideline for reporting systematic reviews. 10.1136/bmj.n71

[20] Higgins JPT, Thomas J, Chandler J, Cumpston M, Li T, Page MJ WV (2019) Cochrane handbook for systematic reviews of interventions version 6.0. Cochrane, 2019. In: Handbook. https://training.cochrane.org/handbook/current%0Ahttps://training.cochrane.org/handbook/current%0Ahttps://training.cochrane.org/handbook. Accessed 20 Feb 2020

[21] Sterne JAC, Savović J, Page MJ, Elbers RG, Blencowe NS, Boutron I, Cates CJ, Cheng HY, Corbett MS, Eldridge SM, Emberson JR, Hernán MA, Hopewell S, Hróbjartsson A, Junqueira DR, Jüni P, Kirkham JJ, Lasserson T, Li T, McAleenan A, Reeves BC, Shepperd S, Shrier I, Stewart LA, Tilling K, White IR, Whiting PF, Higgins JPT (2019). RoB 2: a revised tool for assessing risk of bias in randomised trials. BMJ.

[22] Sterne JA, Hernán MA, Reeves BC, Savović J, Berkman ND, Viswanathan M, Henry D, Altman DG, Ansari MT, Boutron I, Carpenter JR, Chan AW, Churchill R, Deeks JJ, Hróbjartsson A, Kirkham J, Jüni P, Loke YK, Pigott TD, Ramsay CR, Regidor D, Rothstein HR, Sandhu L, Santaguida PL, Schünemann HJ, Shea B, Shrier I, Tugwell P, Turner L, Valentine JC, Waddington H, Waters E, Wells GA, Whiting PF, Higgins JP (2016). ROBINS-I: a tool for assessing risk of bias in non-randomised studies of interventions. BMJ.

[23] Guyatt G, Oxman AD, Akl EA, Kunz R, Vist G, Brozek J, Norris S, Falck-Ytter Y, Glasziou P, Debeer H, Jaeschke R, Rind D, Meerpohl J, Dahm P, Schünemann HJ (2011). GRADE guidelines: 1. Introduction—GRADE evidence profiles and summary of findings tables. J Clin Epidemiol.

[24] Review Manager (RevMan) [Computer program]. Version 5.3. Copenhagen: The Nordic Cochrane Centre, The Cochrane Collaboration 2014. (2014) RevMan 5 | Cochrane Community. Cochrane Community

[25] Kitano S, Iso Y, Tomikawa M, Moriyama M, Sugimachi K (1993). A prospective randomized trial comparing pneumoperitoneum and U-shaped retractor elevation for laparoscopic cholecystectomy. Surg Endosc.

[26] Alijani A, Hanna GB, Cuschieri A (2004). Abdominal wall lift versus positive-pressure capnoperitoneum for laparoscopic cholecystectomy: randomized controlled trial. Ann Surg.

[27] Uen YH, Liang AI, Lee HH (2002). Randomized comparison of conventional carbon dioxide insufflation and abdominal wall lifting for laparoscopic cholecystectomy. J Laparoendosc Adv Surg Tech.

[28] Galizia G, Prizio G, Lieto E, Castellano P, Pelosio L, Imperatore V, Ferrara A, Pignatelli C (2001). Hemodynamic and pulmonary changes during open, carbon dioxide pneumoperitoneum, and abdominal wall-lifting cholecystectomy: a prospective, randomized study. Surg Endosc.

[29] Uen YH, Chen Y, Kuo CY, Wen KC, Koay LB (2007). Randomized trial of low-pressure carbon dioxide-elicited pneumoperitoneum versus abdominal wall lifting for laparoscopic cholecystectomy. J Chinese Med Assoc.

[30] Guido RS, Brooks K, McKenzie R, Gruss J, Krohn MA (1998). A randomized, prospective comparison of pain after gasless laparoscopy and traditional laparoscopy. J Am Assoc Gynecol Laparosc.

[31] Talwar N, Pusuluri R, Arora MP, Pawar M (2006). Randomized controlled trial of conventional carbon dioxide pneumoperitoneum versus gasless technique for laparoscopic cholecystectomy. JK Sci.

[32] Cravello L, D’Ercole C, Roger V, Samson D, Blanc B (1999). Laparoscopic surgery in gynecology: randomized prospective study comparing pneumoperitoneum and abdominal wall suspension. Eur J Obstet Gynecol Reprod Biol.

[33] Goldberg JM, Maurer WG (1997). A randomized comparison of gasless laparoscopy and CO2 pneumoperitoneum. Obstet Gynecol.

[34] Wang JJ, Yang F, Gao T, Li L, Xia H, Li HF (2011). Gasless laparoscopy versus conventional laparoscopy in uterine myomectomy: a single-centre randomized trial. J Int Med Res.

[35] Egawa H, Morita M, Yamaguchi S, Nagao M, Iwasaki T, Hamaguchi S, Kitajima T, Minami J (2006). Comparison between intraperitoneal CO2 insufflation and abdominal wall lift on QT dispersion and rate-corrected QT dispersion during laparoscopic cholecystectomy. Surg Laparosc Endosc Percutaneous Tech.

[36] Ge B, Zhao H, Chen Q, Jin W, Liu L, Huang Q (2014). A randomized comparison of gasless laparoscopic appendectomy and conventional laparoscopic appendectomy. World J Emerg Surg.

[37] Han C, Ding Z, Fan J, Sun J, Qian Y (2012). Comparison of the stress response in patients undergoing gynecological laparoscopic surgery using carbon dioxide pneumoperitoneum or abdominal wall-lifting methods. J Laparoendosc Adv Surg Tech.

[38] Woo WK, Hae M, Jeon SCP, Sang KL, Sung WC (2002). Comparison of immune preservation between CO2 pneumoperitoneum and gasless abdominal lift laparoscopy. JSLS.

[39] Koivusalo AM, Kellokumpu I, Lindgren L (1996). Gasless laparoscopic cholecystectomny: comparison of postoperative recovery with conventional technique. Br J Anaesth.

[40] Koivusalo AM, Kellokumpu I, Lindgren L (1997). Postoperative drowsiness and emetic sequelae correlate to total amount of carbon dioxide used during laparoscopic cholecystectomy. Surg Endosc.

[41] Koivusalo AM, Kellokumpu I, Ristkari S, Lindgren L (1997). Splanchnic and renal deterioration during and after laparoscopic cholecystectomy: a comparison of the carbon dioxide pneumoperitoneum and the abdominal wall lift method. Anesth Analg.

[42] Koivusalo AM, Kellokumpu I, Scheinin M, Tikkanen I, Halme L, Lindgren L (1996). Randomized comparison of the neuroendocrine response to laparoscopic cholecystectomy using either conventional or abdominal wall lift techniques. Br J Surg.

[43] Koivusalo AM, Kellokumpu I, Scheinin M, Tikkanen I, Mäkisalo H, Lindgren L (1998). A comparison of gasless mechanical and conventional carbon dioxide pneumoperitoneum methods for laparoscopic cholecystectomy. Anesth Analg.

[44] Koivusalo AM, Pere P, Valjus M, Scheinin T (2008). Laparoscopic cholecystectomy with carbon dioxide pneumoperitoneum is safe even for high-risk patients. Surg Endosc Other Interv Tech.

[45] Larsen JF, Ejstrud P, Kristensen JU, Svendsen F, Redke F, Pedersen V (2001). Randomized comparison of conventional and gasless laparoscopic cholecystectomy: operative technique, postoperative course, and recovery. J Gastrointest Surg.

[46] Larsen JF, Ejstrud P, Svendsen F, Redke F, Pedersen V, Rahr HB (2001). Randomized study of coagulation and fibrinolysis during and after gasless and conventional laparoscopic cholecystectomy. Br J Surg.

[47] Larsen JF, Ejstrud P, Svendsen F, Pedersen V, Redke F (2002). Systemic response in patients undergoing laparoscopic cholecystectomy using gasless or carbon dioxide pneumoperitoneum: a randomized study. J Gastrointest Surg.

[48] Larsen JF, Svendsen FM, Pedersen V (2004). Randomized clinical trial of the effect of pneumoperitoneum on cardiac function and haemodynamics during laparoscopic cholecystectomy. Br J Surg.

[49] Lindgren L, Koivusalo AM, Kellokumpu I (1995). Conventional pneumoperitoneum compared with abdominal wall lift for laparoscopic cholecystectomy. Br J Anaesth.

[50] Li SH, Deng J, Huang FT, Gan XW, Cao YG (2014). Impact of gasless laparoscopy on circulation, respiration, stress response, and other complications in gynecological geriatrics. Int J Clin Exp Med.

[51] Meijer DW, Rademaker BPM, Schlooz S, Bemelman WA, de Wit LT, Bannenberg JJG, Stijnen T, Gouma DF (1997). Laparoscopic cholecystectomy using abdominal wall retraction. Surg Endosc.

[52] Ninomiya K, Kitano S, Yoshida T, Bandoh T, Baatar D, Matsumoto T (1998). Comparison of pneumoperitoneum and abdominal wall lifting as to hemodynamics and surgical stress response during laparoscopic cholecystectomy. Surg Endosc.

[53] Ogihara Y, Isshiki A, Kindscher JD, Goto H (1999). Abdominal wall lift versus carbon dioxide insufflation for laparoscopic resection of ovarian tumors. J Clin Anesth.

[54] Ortiz-Oshiro E, Mayol J, Aparicio Medrano JC, Rabadan Ruiz L, Sanjuan Garcia MA, Alvarez Fdez-Represa J (2001). Gasless laparoscopic cholecystectomy is not more time-consuming. Surg Endosc.

[55] Ortiz-Oshiro E, Mayol J, Aparicio Medrano JC, Sanjuan Garcia MA, Alvarez Fernández-Represa J (2001). Lactate metabolism during laparoscopic cholecystectomy: comparison between CO2 pneumoperitoneum and abdominal wall retraction. World J Surg.

[56] Schulze S, Lyng KM, Bugge K, Perner A, Bendtsen A, Thorup J, Nielsen HJ, Rasmussen V, Rosenberg J (1999). Cardiovascular and respiratory changes and convalescence in laparoscopic colonic surgery. Comparison between carbon dioxide pneumoperitoneum and gasless laparoscopy. Arch Surg.

[57] Sesti F, Capobianco F, Capozzolo T, Pietropolli A, Piccione E (2008). Isobaric gasless laparoscopy versus minilaparotomy in uterine myomectomy: a randomized trial. Surg Endosc Other Interv Tech.

[58] Sietses C, Von Blomberg ME, Eijsbouts QAJ, Beelen RHJ, Berends FJ, Cuesta MA (2002). The influence of CO2 vs helium insufflation or the abdominal wall lifting technique on the systemic immune response. Surg Endosc Other Interv Tech.

[59] Tan J, Sun Y, Zhong B, Dai H, Wang D (2009). A randomized, controlled study comparing minilaparotomy versus isobaric gasless laparoscopic assisted minilaparotomy myomectomy for removal of large uterine myomas: Short-term outcomes. Eur J Obstet Gynecol Reprod Biol.

[60] Uemura N, Nomura M, Inoue S, Endo J, Kishi S, Saito K, Ito S, Nakaya Y (2002). Changes in hemodynamics and autonomic nervous activity in patients undergoing laparoscopic cholecystectomy: differences between the pneumoperitoneum and abdominal wall-lifting method. Endoscopy.

[61] Vázquez-Rosales MA, Sánchez-Aguilar JM, Hernández-Sierra F, Vázquez-Rosales G, Mandeville PB, Tapia-Pérez JH, Sánchez-Reyna M, Gordillo-Moscoso AA (2010). Experience with a new design of endoretractor for gasless laparoscopic cholecystectomy. Surg Laparosc Endosc Percutaneous Tech.

[62] Vezakis A, Davides D, Gibson JS, Moore MR, Shah H, Larvin M, McMahon MJ (1999). Randomized comparison between low-pressure laparoscopic cholecystectomy and gasless laparoscopic cholecystectomy. Surg Endosc.

[63] Vofsi O, Barak M, Moscovici R, Bustan M, Katz Y (2004). Cardiorespiratory parameters during conventional or gasless gynecological laparoscopy under general or regional anesthesia. Med Sci Monit.

[64] Yoshida T, Kobayashi E, Suminaga Y, Yamauchi H, Kai T, Toyama N, Kiyozaki H, Fujimura A, Miyata M (1997). Hormone-cytokine response: pneumoperitoneum vs abdominal wall-lifting in laparoscopic cholecystectomy. Surg Endosc.

[65] Zaporozhchenko BS, Kolodiǐ VV, Gorbunov AA, Zaporozhchenko MB, Kirpichnikova EP (2013). Experience of simultaneous laparoscopic cholecystectomy and gynecologic operations performance in conditions of withous gas laparoscopy in patients of high operation-anesthesiology risk. Klin Khir.

[66] Zaporozhchenko BS, Kolodiy VV, Gorbunov AA, Zaporozhchenko MB, Muravyev PT, Kholodov IG (2017). Lifting laparoscopy in simultant surgery. Klin khirurhiia.

[67] Chang TC, Chen CC, Wang MY, Yang CY, Lin MT (2011). Gasless laparoscopy-assisted distal gastrectomy for early gastric cancer: analysis of initial results. J Laparoendosc Adv Surg Tech.

[68] Chou TH, Wu MH, Wang MY, Yang CY, Lai PS, Lin MT, Lee PH (2008). Gasless laparoscopy-assisted subtotal gastrectomy for early gastric cancer: a novel minimally invasive surgery. J Gastrointest Surg.

[69] Fukushima R, Kawamura YJ, Saito H, Saito Y, Hashiguchi Y, Sawada T, Muto T (1996). Interleukin-6 and stress hormone responses after uncomplicated gasless laparoscopic-assisted and open sigmoid colectomy. Dis Colon Rectum.

[70] Jiang JK, Chen WS, Wang SJ, Lin JK (2010). A novel lifting system for minimally accessed surgery: a prospective comparison between “Laparo-V” gasless and CO2 pneumoperitoneum laparoscopic colorectal surgery. Int J Colorectal Dis.

[71] Kurauchi N, Yonekawa M, Kurokawa Y, Tamiya Y, Nakamura S, Nagai H, Obara Y, Terada H, Yamada H, Yamada Y, Nakamura H, Hashimoto D, Mori T, Yamakawa T, Hagiwara M, Ishihara H, Nishii H, Sekimoto M, Urushihara T, Ota J, Taniguchi Y, Yamaguchi H, Kitano S (1999). Comparison between CO2 insufflation and abdominal wall lift in laparoscopic cholecystectomy: a prospective multiinstitutional study in Japan. Surg Endosc.

[72] Lee PC, Lai PS, Yang CY, Chen CN, Lai IR, Lin MT (2013). A gasless laparoscopic technique of wide excision for gastric gastrointestinal stromal tumor versus open method. World J Surg Oncol.

[73] Liao CH, Kuo IM, Fu CY, Chen CC, Yang SJ, Ouyang CH, Wang SY, Chen SW, Hsu YP, Kang SC (2014). Gasless laparoscopic assisted surgery for abdominal trauma. Injury.

[74] Nanashima A, Yamaguchi H, Tsuji T, Yamaguchi E, Sawai T, Yasutake T, Nakagoe T, Ayabe H (1998). Physiologic stress responses to laparoscopic cholecystectomy: a comparison of the gasless and pneumoperitoneal procedures. Surg Endosc.

[75] Palomba S, Zupi E, Falbo A, Russo T, Marconi D, Zullo F (2010). New tool (Laparotenser) for gasless laparoscopic myomectomy: a multicenter-controlled study. Fertil Steril.

[76] Tintara H, Choobun T (2004). Laparoscopic adnexectomy for benign tubo-ovarian disease using abdominal wall lift: a comparison to laparotomy. Int J Gynecol Obstet.

[77] Tintara H, Choobun T, Geater A (2003). Gasless laparoscopic hysterectomy: a comparative study with total abdominal hysterectomy. J Obstet Gynaecol Res.

[78] Ülker K, Hüseyinoǧlu Ü (2013). Comparison of tubal sterilization procedures performed by keyless abdominal rope-lifting surgery and conventional CO2 laparoscopy: a case controlled clinical study. Sci World J.

[79] Ülker K, Hüseyinoğlu Ü, Çiçek M (2015). Early postoperative pain after keyless abdominal rope-lifting surgery. J Soc Laparoendosc Surg.

[80] Ülker K, Hüseyinoǧlu Ü, Kiliç N (2013). Management of benign ovarian cysts by a novel, gasless, single-incision laparoscopic technique: keyless abdominal rope-lifting surgery (KARS). Surg Endosc.

[81] Wang Y, Cui H, Zhao Y, Wang ZQ (2009). Gasless laparoscopy for benign gynecological diseases using an abdominal wall-lifting system. J Zhejiang Univ Sci B.

[82] Wu JM, Yang CY, Wang MY, Wu MH, Lin MT (2010). Gasless laparoscopy-assisted versus open resection for gastrointestinal stromal tumors of the upper stomach: preliminary results. J Laparoendosc Adv Surg Tech.

[83] Zhong CX, Wu JX, Liang JX, Wu QH (2012). Laparoscopic and gasless laparoscopic sigmoid colon vaginoplasty in women with vaginal agenesis. Chin Med J.

[84] Cheng J, Pullenayegum E, Marshall JK, Iorio A, Thabane L (2016). Impact of including or excluding both-armed zero-event studies on using standard meta-analysis methods for rare event outcome: a simulation study. BMJ Open.

[85] Weiser TG, Forrester JA, Negussie T (2019). Implementation science and innovation in global surgery. BJS.

[86] Mcculloch P, Altman DG, Campbell B, Flum DR, Glasziou P, Marshall JC, Nicholl J (2009). No surgical innovation and evaluation 3 no surgical innovation without evaluation: the IDEAL recommendations. Lancet.

[87] Farrow NE, Commander SJ, Reed CR, Mueller JL, Gupta A, Loh AHP, Sekabira J, Fitzgerald TN (2020). Laparoscopic experience and attitudes toward a low-cost laparoscopic system among surgeons in East, Central, and Southern Africa: a survey study. Surg Endosc.

[88] Alfa-Wali M, Osaghae S (2017). Practice, training and safety of laparoscopic surgery in low and middle-income countries. World J Gastrointest Surg.

[89] Sesti F, Pietropolli A, Sesti FF, Piccione E (2013). Gasless laparoscopic surgery during pregnancy: evaluation of its role and usefulness. Eur J Obstet Gynecol Reprod Biol.

[90] Rosenbaum AJ, Maine RG (2019). Improving access to laparoscopy in low-resource settings. Ann Glob Heal.

[91] Dindo D, Demartines N, Clavien P-A (2004). Classification of surgical complications: a new proposal with evaluation in a cohort of 6336 patients and results of a survey. Ann Surg.

