# Supplementary material for: Clinical effectiveness of gasless laparoscopic surgery for abdominal conditions: systematic review and meta-analysis
Source: Surg Endosc. 2021 Aug 16;35(12):6427–37. doi: 10.1007/s00464-021-08677-7 (PMC8599349; doi:10.1007/s00464-021-08677-7)
Supplement: Supplementary file 1 — Supplementary file1 (DOCX 163 kb) [file 464_2021_8677_MOESM1_ESM.docx]

**Clinical effectiveness of gasless laparoscopic surgery for abdominal conditions: systematic review and meta-analysis**

N Aruparayil MD^1^, W Bolton MBChB^1^, A Mishra MD^2^, L Bains MD^2^, J Gnanaraj MCh^3^, R King PhD^4^, Professor T Ensor PhD^4^, N King MSc^5^, Professor D Jayne MD^1^, B Shinkins PhD^5^

^1^Leeds Institute of Medical Research at St. James’s, University of Leeds, UK

^2^Maulana Azad Medical College, Delhi, India

^3^Karunya University, Coimbatore, India

^4^Nuffield Centre for International Health and Development, Leeds Institute of Health Sciences, University of Leeds, UK

^5^Academic Unit of Health Economics, Leeds Institute of Health Sciences, University of Leeds, UK

KEYWORDS

*Gasless laparoscopy, abdominal wall lift, LMIC, low resource setting, clinical effectiveness, open surgery, general surgery, gynaecological surgery*

Address of Correspondence:

Mr Noel Aruparayil

NIHR Global Health Research Group – Surgical Technologies

Clinical Sciences Building

Level 7, Room 7.19

Leeds

LS9 7TF

[n.k.aruparayil@leeds.c.uk](mailto:n.k.aruparayil@leeds.c.uk)

+447540775214

**TABLES (supplement)**

**TABLE 1: Included RCTs - General and Gynaecology procedures**

| Author | Year | Country | Type | Follow up period | Organ(s) | Comparison | AWL device | Number of participants |
| --- | --- | --- | --- | --- | --- | --- | --- | --- |
| Alijani et al.[26] | 2004 | UK | RCT | 6 weeks | Gallbladder | Gasless vs Conventional | Laparotensor, Lucini, Milan | 40 |
| Cravello et al.[37] | 1998 | France | RCT | Not recorded | Adnexa | Gasless vs Conventional | Laparolift, California, USA | 103 |
| Egawa et al.[45] | 2006 | Japan | RCT | Not recorded | Gallbladder | Gasless vs Conventional | Laparotensor, Lucini, Milan | 30 |
| Galizia et a.[28] | 2001 | Italy | RCT | Not recorded | Gallbladder | Gasless vs open vs Conventional | Laparotensor, Lucini, Milan | 15 |
| Ge et al.[46] | 2014 | China | RCT | 30 days | Appendix | Gasless vs Conventional | Subcutaneous LiftSystem (Mizuho Co., Tokyo, Japan | 100 |
| Goldberg et al.[38] | 1997 | USA | RCT | 2 weeks | Adnexa, appendix | Gasless vs Conventional | Laparolift | 51 |
| Guido et al. [30] | 1998 | USA | RCT | 14 days | Adnexa | Gasless vs Conventional | Laparolift | 77 |
| Han et al. [47] | 2012 | China | RCT | 3 days | Uterus | Gasless vs Conventional | Laparolift | 57 |
| Kim et al. [48] | 2002 | S Korea | RCT | No info | Gallbladder | Gasless vs Conventional | Kim’s lifter, Sejong, S Korea | 100 |
| Kitano et al. [25] | 1993 | Japan | RCT | 1 year | Gallbladder | Gasless vs Conventional | U Shaped Retractor | 83 |
| *Koivusalo et al. [49](1a) | 1996 | Finland | RCT | Not recorded | Gallbladder | Gasless vs Conventional | Laparolift | 26 |
| Koivusalo et al.[50] (a) | 1997 | Finland | RCT | Not recorded | Gallbladder | Gasless vs Conventional | AWL Lift - Cuscheri | 25 |
| Koivusalo et al. [51] (b) | 1997 | Finland | RCT | Not recorded | Gallbladder | Gasless vs Conventional | Laparolift | 30 |
| Koivusalo et al . [52](b) | 1996 | Finland | RCT | Not recorded | Gallbladder | Gasless vs Conventional | AWL Lift - Hoffman’s trocar | 24 |
| *Koivusalo et al.[53](1b) | 1998 | Finland | RCT | Not recorded | Gallbladder | Gasless vs Conventional | Laparolift | 26 |
| Koivusalo et al. [54] | 2008 | Finland | RCT | Not recorded | Gallbladder | Gasless vs Conventional | Laparolift | 20 |
| **Larsen, et al.[55](1a) | 2001 | Denmark | RCT | Not recorded | gallbladder | gasless vs conventional | Laparotensor, Lucini, Milan | 50 |
| **Larsen, et al.[56](1b) | 2001 | Denmark | RCT | Not recorded | gallbladder | gasless vs conventional | Laparotensor, Lucini, Milan | 50 |
| **Larsen, et al.[57](1c) | 2002 | Denmark | RCT | 14 days | gallbladder | gasless vs conventional | Laparotensor, Lucini, Milan | 50 |
| Larsen, et al.[58] | 2004 | Denmark | RCT | Not recorded | gallbladder | gasless vs conventional | Laparotensor, Lucini, Milan | 50 |
| Lindgren, et al. [59] | 1995 | Finland | RCT | Not recorded | gallbladder | gasless vs conventional | AWL Liift - Hoffman's trocar | 25 |
| Li, S. H et al.[60] | 2014 | China | RCT | Not recorded | Adnexa | gasless vs conventional | kirschner wire - lifting | 40 |
| Meijer et al.[61] | 1997 | Netherlands | RCT | Not recorded | gallbladder | gasless vs conventional | Laparolift | 20 |
| Mishra et al.[13] | 2020 | India | RCT | 7 days | Gallbladder, appendix | Gasless vs conventional | Staan Lift | 100 |
| Ninomiya et al. (Kitano) [62] | 1998 | Japan | RCT | Not recorded | gallbladder | gasless vs conventional | U shaped retractor | 20 |
| Ogihara, et al. [63] | 1999 | USA | RCT | Not recorded | ovary | gasless vs conventional | Lifting arm - Mizuho Medical, Tokyo | 12 |
| ***Ortiz-Oshiro, et al.[64] | 2001 | Spain | RCT | Not recorded | gallbladder | gasless vs conventional | AWL Aesculap | 34 |
| ***Ortiz-Oshiro, et al.[65] | 2001 | Spain | RCT | Not recorded | gallbladder | gasless vs conventional | AWL Aesculap | 34 |
| Schulze, et al.[66] | 1999 | Denmark | RCT | 30 days | colon | gasless vs conventional | Laparolift | 22 |
| Sesti, et al.[67] | 2008 | Italy | RCT | 30 days | myoma - uterus | gasless vs open | Laparotensor, Lucini, Milan | 100 |
| Sietses, et al.[68] | 2002 | Netherlands | RCT | Not recorded | gallbladder | gasless vs conventional | AWL Aesculap - Variolift, Germany | 33 |
| Talwar, et al.[31] | 2006 | India | RCT | Not recorded | gallbladder | gasless vs conventional | Laparolift | 40 |
| Tan, et al.[69] | 2009 | China | RCT | 2 days | myoma - uterus | gasless vs open | Gasless lifting device - Japan Daoke Co | 80 |
| Uemura, et al. [70] | 2002 | Japan | RCT | Not recorded | gallbladder | gasless vs conventional | Lifting arm - Mizuho Medical, Tokyo | 33 |
| Uen, et al.[27] | 2002 | Taiwan | RCT | Not recorded | gallbladder | gasless vs conventional | AWL Hashimoto | 95 |
| Uen, et al.[29] | 2007 | Taiwan | RCT | Not recorded | gallbladder | gasless vs conventional | AWL Hashimoto | 79 |
| Vazquez-Rosales, et al. [71] | 2010 | Mexico | RCT | Not recorded | gallbladder | gasless vs conventional | Modified AWL retractor | 22 |
| Vezakis, et al.[72] | 1999 | England | RCT | 6 weeks | gallbladder | gasless vs conventional | Laparotensor, Lucini, Milan | 42 |
| Vofsi, et al.[73] | 2004 | Israel | RCT | Not recorded | Adnexa/uterus | conventional vs gasless (general) vs gasless (regional) | Unspecified | 24 |
| Wang, et al.[39] | 2011 | China | RCT | Not recorded | myoma - uterus | gasless vs conventional | Laparolift/ Laparotensor, Lucini, Milan | 384 |
| Yoshida, et al.[74] | 1997 | Japan | RCT | Not recorded | gallbladder | gasless vs conventional | Kirschner wires | 17 |

*Koivusalo, **Larsen and ***Ortiz-Oshiro - same population, different studies

**Table 2: Non-RCT comparative cohort studies - General Surgery and Gynaecology**

| Author | YEAR | Country | TYPE | Organs | Type of comparison | Device | Participants |
| --- | --- | --- | --- | --- | --- | --- | --- |
| Akira, et al.[15] | 1999 | Japan | Prospective cohort, retrospective control | ovaries | gasless vs laparotomy | Lifting arm - Mizuho Medical, Tokyo | 35 |
| Chang, et al.[75] | 2011 | Taiwan | Prospective cohort | Stomach | gasless vs conventional | Kents clamp and retaining retractors | 75 |
| Chou, et al.[76] | 2008 | Taiwan | Prospective cohort | Stomach | gasless vs conventional | Kents clamp and retaining retractors | 41 |
| Fukushima, et al.[77] | 1996 | Japan | Prospective cohort | Colon | gasless vs open | Lifting arm - Mizuho Medical, Tokyo | 14 |
| Huang, et al.[16] | 2010 | Taiwan | Retrospective cohort | small bowel | gasless vs open | Kents clamp and retaining retractors | 25 |
| Jiang, et al.[78] | 2010 | Taiwan | Prospective cohort | Colon | lap vs open vs gasless | Laparo - V | 59 |
| Kim, et al.[14] | 2020 | S Korea | Retrospective cohort | uterus | gasless vs conventional | J shaped retractor | 80 |
| Kurauchi et al. [79] | 1999 | Japan | Prospective cohort | Gallbladder | gasless vs conventional | subcutaneous lift Nagai’s procedure, Hashimoto’s method, and peritoneal lift Kitano’s technique Nakamura’s fishing-rod type, Nishii’s method and Laparoliftt | 155 |
| Lee, et al.[80] | 2013 | Taiwan | Retrospective cohort | Stomach | gasless vs conventional | self-sustained retractor | 62 |
| Liao, et al.[81] | 2014 | Taiwan | Retrospective cohort | Multi organ | gasless vs open | Abdolift, Germany | 45 |
| Nanashima, et al.[82] | 1998 | Japan | Prospective cohort | Gallbladder | gasless vs conventional | Laparolift | 27 |
| Palomba, et al.[83] | 2010 | Italy | Prospective cohort, retrospective control | Myoma - Uterus | gasless vs conventional | Laparotensor | 60 |
| Tintara [84] | 2004 | Thailand | Prospective cohort, retrospective control | Adnexa | Gasless vs open | Modified lifting device | 68 |
| Tintara, et al.[85] | 2003 | Thailand | Prospective cohort | Uterus | gasless vs open | Laparolift | 62 |
| Ulker, et al. [86] | 2013 | Turkey | Prospective cohort | Fallopian tube | gasless vs conventional | KRAS^*^ | 71 |
| Ulker, et al.[87] | 2015 | Turkey | Prospective cohort | ovaries | gasless vs conventional | KRAS^*^ | 77 |
| Ulker, et al.[88] | 2013 | Turkey | Prospective cohort | ovaries | gasless vs conventional | KRAS^*^ | 55 |
| Wang Y, et al.[89] | 2009 | China | Prospective cohort | uterus and adnexa | gasless vs conventional | Lifting arm - Mizuho Medical, Tokyo | 76 |
| Wu, et al.[90] | 2010 | Taiwan | Prospective cohort | upper GI | gasless vs open | Kents clamp and retaining retractors | 28 |
| Zaporozhchenko, et al.[32] | 2013 | Ukraine | Prospective cohort | gallbladder and adnexa | gasless vs conventional | Laparolift | 67 |
| Zaporozhchenko, et al.[33] | 2017 | Ukraine | Prospective cohort | gallbladder and adnexa | gasless vs conventional | Laparolift | 84 |
| Zhong, L. Bu, et al.[91] | 2012 | China | Prospective cohort | sigmoid/vagina | gasless vs conventional | Subcutaneous LiftSystem (Mizuho Co., Tokyo, Japan | 119 |

KRAS^*^ - Keyless abdominal rope-lifting surgery

**Table 3: RCT RoB-2 result level assessment for each study across 5 domains**

| Study | Randomization process | Deviations from intended interventions | Missing outcome data | Measurement of the outcome | Selection of the reported result | Overall |
| --- | --- | --- | --- | --- | --- | --- |
| Alijani, 2004 |  |  |  |  |  |  |
| Intraop | Low | Low | Low | High | Some concerns | High |
| Conversion | Low | Low | Low | Low | Low | Low |
| Overal CL | Low | Low | Low | High | Some concerns | High |
| Op time | Low | Low | Low | Low | Low | Low |
| Cravello, 1999 |  |  |  |  |  |  |
| intraop | Some concerns | Low | Low | High | Some concerns | High |
| overal CL | Some concerns | Low | Low | High | Some concerns | High |
| conversion | Some concerns | Low | Low | Low | Low | Some concerns |
| LoS | Some concerns | Low | Low | Low | Low | Some concerns |
| Egawa, 2006 |  |  |  |  |  |  |
| Op time | Some concerns | Low | Low | Low | Low | Some concerns |
| Galazia, 2001 |  |  |  |  |  |  |
| intraop | Low | Low | Low | High | Some concerns | High |
| Overal CL | Low | Low | Low | High | Some concerns | High |
| op time | Low | Low | Low | Low | Low | Low |
| LoS | Low | Low | Low | Low | Low | Low |
| Ge, 2014 |  |  |  |  |  |  |
| overal CL | High | Low | Low | Some concerns | Low | High |
| op time | High | Low | Low | Low | Low | High |
| conversion | High | Low | Low | Low | Low | High |
| LoS | High | Low | Low | Low | Low | High |
| Goldberg, 1997 |  |  |  |  |  |  |
| Conversion | Low | Low | Low | Low | Low | Low |
| Overal CL | Low | Low | Low | High | Some concerns | High |
| Op time | Low | Low | Low | Low | Low | Low |
| Guido, 1998 |  |  |  |  |  |  |
| Intraop | Low | Low | Low | High | Some concerns | High |
| overal CL | Low | Low | Low | High | Some concerns | High |
| Han, 2012 |  |  |  |  |  |  |
| op time | Low | Low | Low | Low | Low | Low |
| Kim, 2002 |  |  |  |  |  |  |
| Op time | Some concerns | Low | Low | Low | Low | Some concerns |
| Kitano, 1993 |  |  |  |  |  |  |
| Intraop | Some concerns | Low | Low | High | Some concerns | High |
| Conversion | Some concerns | Low | Low | Low | Low | Some concerns |
| Overal CL | Some concerns | Low | Low | High | Some concerns | High |
| Koivusalo, 1997b |  |  |  |  |  |  |
| op time | Some concerns | Low | Low | Low | Low | Some concerns |
| Koivusalo, 19961a |  |  |  |  |  |  |
| op time | Some concerns | Low | Low | Low | Low | Some concerns |
| Koivusalo, 19961b |  |  |  |  |  |  |
| overal CL | Some concerns | Low | Low | High | Some concerns | High |
| op time | Some concerns | Low | Low | Low | Low | Some concerns |
| Koivusalo, 2008 |  |  |  |  |  |  |
| overal CL | Low | Low | Low | Low | Low | Low |
| conversion | Low | Low | Low | Low | Low | Low |
| op time | Low | Low | Low | Low | Low | Low |
| loS | Low | Low | Low | Low | Low | Low |
| Larsen, 1a2001 |  |  |  |  |  |  |
| overal CL | Low | Low | High | Low | Low | High |
| conversion | Low | Low | Low | Low | Low | Low |
| op time | Low | Low | Low | Low | Low | Low |
| Larsen, 2004 |  |  |  |  |  |  |
| Conversion | Low | Low | Low | Low | Low | Low |
| op time | Low | Low | Low | Low | Low | Low |
| Lindgren, 1995 |  |  |  |  |  |  |
| Op time | Some concerns | Low | Low | Low | Low | Some concerns |
| Li SH, 2014 |  |  |  |  |  |  |
| Op time | Some concerns | Low | Low | Low | Low | Some concerns |
| Meijer, 1997 |  |  |  |  |  |  |
| Op time | Some concerns | Low | Low | Low | Low | Some concerns |
| Conversion | Some concerns | Low | Low | Low | Low | Some concerns |
| Mishra, 2020 |  |  |  |  |  |  |
| overal CL | Low | Low | Low | High | Low | High |
| op time | Low | Low | Low | Low | Low | Low |
| conversion | Low | Low | Low | Low | Low | Low |
| Ninomiya, 1998 |  |  |  |  |  |  |
| op time | Some concerns | Low | Low | Low | Low | Some concerns |
| Ogihara, 1999 |  |  |  |  |  |  |
| op time | Some concerns | Low | Low | Low | Low | Some concerns |
| Oritz-Oshiro, 1a 2001 |  |  |  |  |  |  |
| op time | Low | Some concerns | Low | Low | Low | Some concerns |
| Schulze, 1999 |  |  |  |  |  |  |
| overal CL | Some concerns | High | Low | High | Some concerns | High |
| conversion | Some concerns | Low | Low | Low | Low | Some concerns |
| op time | Some concerns | Low | Low | Low | Low | Some concerns |
| Sesti, 2008 |  |  |  |  |  |  |
| conversion | Low | Low | Low | Low | Low | Low |
| op time | Low | Low | Low | Low | Low | Low |
| Sietses, 2002 |  |  |  |  |  |  |
| intraop | Some concerns | Low | Low | High | Some concerns | High |
| op time | Some concerns | Low | Low | Low | Low | Some concerns |
| Talwar, 2006 |  |  |  |  |  |  |
| overal CL | Low | Low | Low | Low | Low | Low |
| conversion | Low | Low | Low | Low | Low | Low |
| op time | Low | Low | Low | Low | Low | Low |
| Tan, 2009 |  |  |  |  |  |  |
| intraop | Some concerns | Low | Low | Low | Low | Some concerns |
| conversion | Some concerns | Low | Low | Low | Low | Some concerns |
| op time | Some concerns | Low | Low | Low | Low | Some concerns |
| LoS | Some concerns | Low | Low | Low | Low | Some concerns |
| Uemura, 2002 |  |  |  |  |  |  |
| Op time | Some concerns | Low | Low | Low | Low | Some concerns |
| Uen, 2002 |  |  |  |  |  |  |
| intraop | Low | Low | Low | Low | Low | Low |
| overal CL | Low | Low | Low | Low | Low | Low |
| conversion | Low | Low | Low | Low | Low | Low |
| op time | Low | Low | Low | Low | Low | Low |
| loS | Low | Low | Low | Low | Low | Low |
| Uen, 2007 |  |  |  |  |  |  |
| intraop | Low | Low | Low | Low | Low | Low |
| overal CL | Low | Low | Low | Low | Low | Low |
| conversion | Low | Low | Low | Low | Low | Low |
| op time | Low | Low | Low | Low | Low | Low |
| loS | Low | Low | Low | Low | Low | Low |
| Vazquez-Rosales, 2010 |  |  |  |  |  |  |
| conversion | Some concerns | Low | Low | Low | Low | Some concerns |
| op time | Some concerns | Low | Low | Low | Low | Some concerns |
| loS | Some concerns | Low | Low | Low | Low | Some concerns |
| Vezakis, 1999 |  |  |  |  |  |  |
| overal CL | Low | High | Low | Low | Low | High |
| conversion | Low | Low | Low | Low | Low | Low |
| op time | Low | Low | Low | Low | Low | Low |
| Vofsi, 2004 |  |  |  |  |  |  |
| LoS | Low | Low | Low | Low | Low | Low |
| wang, 2011 |  |  |  |  |  |  |
| conversion | Low | Low | Low | Low | Low | Low |
| LoS | Low | Low | Low | Low | Low | Low |
| Yoshida, 1997 |  |  |  |  |  |  |
| op time | Low | Low | Low | Low | Low | Low |
| LoS | Low | Low | Low | Low | Low | Low |

**(Results reported as ‘not estimable’ in the forest plot have been excluded in the risk of bias assessment using RoB-2)**

**Table 4: Non-RCT (Comparative cohort studies) ROBINS-I result level assessment for each study across 7 domains**

| Name | Type of study | Bias due to confounding | Bias in selection of participants into the study | Bias in classification of interventions | Bias due to deviations from intended interventions | Bias due to missing data | Bias in measurement of outcomes | Bias in selection of the reported result | Overall bias |
| --- | --- | --- | --- | --- | --- | --- | --- | --- | --- |
| Akira 1999 | Prospective cohort, retrospective control |  |  |  |  |  |  |  |  |
| Ov compl |  | moderate | low | low | low | low | serious | serious | serious |
| Op time |  | moderate | low | low | low | low | low | low | moderate |
| LoS |  | moderate | low | low | low | low | low | low | moderate |
| Chang 2011 | prospective cohort |  |  |  |  |  |  |  |  |
| Ov compl |  | moderate | low | low | low | low | moderate | moderate | moderate |
| op time |  | moderate | low | low | low | low | low | low | moderate |
| LoS |  | moderate | low | low | low | low | low | low | moderate |
| Chou 2008 | prospective cohort |  |  |  |  |  |  |  |  |
| Ov compl |  | low | low | low | low | low | moderate | low | moderate |
| op time |  | low | low | low | low | low | low | low | low |
| LoS |  | low | low | low | low | low | low | low | low |
| Fukushima 1998 | prospective cohort |  |  |  |  |  |  |  |  |
| op time |  | moderate | low | low | low | low | low | low | moderate |
| LoS |  | moderate | low | low | low | low | low | low | moderate |
| Huang 2010 | Retrospective cohort |  |  |  |  |  |  |  |  |
| ov compl |  | moderate | low | low | low | low | low | low | moderate |
| LoS |  | moderate | low | low | low | low | low | low | moderate |
| Jiang 2010 | prospective cohort |  |  |  |  |  |  |  |  |
| Op time |  | moderate | low | low | moderate | low | low | low | moderate |
| conversion |  | serious | low | low | moderate | low | moderate | low | serious |
| LoS |  | serious | low | low | moderate | low | low | low | serious |
| Kim 2020 | retrospective cohort |  |  |  |  |  |  |  |  |
| op time |  | moderate | low | low | low | low | low | low | moderate |
| Kurauchi 1999 | prospective cohort |  |  |  |  |  |  |  |  |
| op time |  | moderate | moderate | moderate | Low | Low | Low | Low | moderate |
| ov compl |  | serious | moderate | moderate | low | low | moderate | moderate | serious |
| conversion |  | serious | moderate | moderate | low | low | low | low | serious |
| LoS |  | serious | moderate | moderate | Low | low | low | low | serious |
| Lee 2013 | retrospective cohort |  |  |  |  |  |  |  |  |
| op time |  | moderate | low | low | low | low | low | low | moderate |
| LoS |  | moderate | low | low | low | low | low | low | moderate |
| Liao 2014 | Retrospective cohort |  |  |  |  |  |  |  |  |
| op time |  | low | low | low | low | low | low | low | low |
| ov compl |  | moderate | low | low | low | low | low | low | moderate |
| conversion |  | moderate | low | low | low | low | low | low | moderate |
| LoS |  | moderate | low | low | low | low | low | low | moderate |
| Nanashima 1998 | prospective, before and after intervention |  |  |  |  |  |  |  |  |
| op time |  | moderate | low | low | low | low | low | low | moderate |
| LoS |  | moderate | low | low | low | low | low | low | moderate |
| Palomba 2010 | Prospective cohort, retrospective control |  |  |  |  |  |  |  |  |
| intraop cl |  | moderate | low | low | low | low | moderate | low | moderate |
| op time |  | low | low | low | low | low | low | low | low |
| ov compl |  | moderate | low | low | low | low | moderate | low | moderate |
| conversion |  | moderate | low | low | low | low | low | low | moderate |
| LoS |  | moderate | low | low | low | low | low | low | moderate |
| Tintara 2003 | prospective, before and after intervention |  |  |  |  |  |  |  |  |
| op time |  | moderate | low | low | low | low | low | low | moderate |
| ov compl |  | serious | moderate | low | low | low | low | low | serious |
| conversion |  | serious | moderate | low | low | low | low | low | serious |
| LoS |  | moderate | low | low | low | low | low | low | moderate |
| Tintara 2004 | Prospective cohort, retrospective control |  |  |  |  |  |  |  |  |
| op time |  | moderate | moderate | low | low | low | low | low | moderate |
| ov compl |  | moderate | moderate | low | low | low | moderate | low | moderate |
| conversion |  | moderate | low | low | low | low | low | low | moderate |
| LoS |  | moderate | low | low | low | low | low | low | moderate |
| Ulker 2015 | prospective, before and after intervention |  |  |  |  |  |  |  |  |
| conversion |  | moderate | low | low | low | low | low | low | moderate |
| op time |  | low | low | low | low | low | low | low | low |
| LoS |  | moderate | low | low | low | low | low | low | moderate |
| Ulker 2013 T | prospective cohort | Low | Low | Low | Low | Low | Low | Low | Low |
| Op time |  | moderate | low | low | low | low | low | low | moderate |
| LoS |  | moderate | low | low | low | low | low | low | moderate |
| Ulker 2013 O | prospective cohort |  |  |  |  |  |  |  |  |
| Op time |  | moderate | low | low | low | low | low | low | moderate |
| LoS |  | moderate | low | low | low | low | low | low | moderate |
| Wang 2009 | prospective cohort |  |  |  |  |  |  |  |  |
| op time |  | moderate | low | low | low | low | low | low | moderate |
| Wu 2010 | prospective cohort |  |  |  |  |  |  |  |  |
| op time |  | moderate | low | low | low | low | low | low | moderate |
| LoS |  | moderate | low | low | low | low | low | low | moderate |
| Zaporozhchenko 2013 | prospective cohort |  |  |  |  |  |  |  |  |
| op time |  | moderate | low | low | low | low | low | low | moderate |
| conversion |  | moderate | low | low | low | low | low | low | moderate |
| ov compl |  | moderate | low | low | low | low | moderate | low | moderate |
| Zaporozhchenko 2017 | prospective cohort |  |  |  |  |  |  |  |  |
| op time |  | moderate | low | low | low | low | low | low | moderate |
| conversion |  | moderate | low | low | low | low | low | low | moderate |
| ov compl |  | moderate | low | low | low | low | low | low | moderate |
| Zhong 2013 | prospective cohort |  |  |  |  |  |  |  |  |
| Op time |  | moderate | low | low | low | low | low | low | moderate |
| Ov compl |  | serious | low | low | low | low | moderate | low | serious |

**(Results reported as ‘not estimable’ in the forest plot have been excluded in the risk of bias assessment using ROBINS-I)**

**Table 5: GRADE assessment for RCT studies comparing Gasless vs conventional surgery**

| **GENERAL SURGERY** | | | | | | | | | | |
| --- | --- | --- | --- | --- | --- | --- | --- | --- | --- | --- |
|  |  | **Certainty assessment** | | | | **Number of patients** | | **Effect** | | **Certainty** |
| **Outcome** | **№ of studies** | **Risk of bias** | **Inconsistency** | **Indirectness** | **Imprecision** | **Gasless** | **Conventional** | **Relative (95% CI)** | **Absolute (95% CI)** | **GRADE** |
| Intraoperative complications | 19 | serious | Not serious | serious | serious | 14/101 (3.5%) | 12/405 (3.0%) | **RR 1.04** (0.45 to 2.40) | **1 more per 1,000** (from 16 fewer to 41 more) | ⨁◯◯◯ VERY LOW^1^ |
| Conversion rate | 12 | serious | Not serious | serious | serious | 23/353 (6.5%) | 16/360 (4.4%) | **RR 1.57** (0.70 to 3.50) | **25 more per 1,000** (from 13 fewer to 111 more) | ⨁⨁◯◯ LOW^2^ |
| Overall complications | 19 | serious | serious | Not serious | serious | 40/416 (9.6%) | 43/413 (10.4%) | **RR 0.89** (0.56 to 1.43) | **11 fewer per 1,000** (from 46 fewer to 45 more) | ⨁◯◯◯ VERY LOW^1^ |
| Operative time | 25 | serious | serious | serious | Not serious | 514 | 532 | - | **MD 8.53 higher** (4.68 higher to 12.38 higher) | ⨁⨁◯◯ LOW^3^ |
| Length of stay | 13 | serious | serious | serious | Not serious | 225 | 227 | - | MD **0.02 lower** (0.52 lower to 0.56 higher) | ⨁◯◯◯ VERY LOW^5^ |
| **GYNAECOLOGY** | | | | | | | | | | |
| Intraoperative complications | 6 | serious | Not serious | serious | serious | 3/315 (1.0%) | 4/321 (1.2%) | **RR 1.04** (0.45 to 2.40) | **0 fewer per 1,000** (from 7 fewer to 17 more) | ⨁◯◯◯ VERY LOW^1^ |
| Conversion rate | 3 | serious | Not serious | serious | serious | 15/264 (5.7%) | 1/270(0.4%) | **RR 11.72** (2.26 to 60.87) | **40 more per 1,000** (from 5 more to 222 more) | ⨁◯◯◯ VERY LOW^5^ |
| Overall complications | 6 | serious | serious | serious | serious | 7/315 (2.2%) | 7/321 (2.2%) | **RR 0.89** (0.56 to 1.43) | **2 fewer per 1,000** (from 10 fewer to 9 more) | ⨁◯◯◯ VERY LOW^5^ |
| Operative time | 6 | serious | Not serious | serious | serious | 146 | 150 | - | MD **0.02 lower** (8.9 lower to 8.86 higher) | ⨁◯◯◯ VERY LOW^5^ |
| Length of stay | 3 | serious | Not serious | serious | serious | 249 | 244 | - | MD **0.93 higher** (0.58 higher to 1.27 higher) | ⨁◯◯◯ VERY LOW^5^ |

**CI:** Confidence interval; **RR:** Risk ratio; **MD:** Mean difference

GRADE Working Group grades of evidence

**High quality:** Further research is very unlikely to change our confidence in the estimate of effect.
**Moderate quality:** Further research is likely to have an important impact on our confidence in the estimate of effect and may change the estimate.
**Low quality:** Further research is very likely to have an important impact on our confidence in the estimate of effect and is likely to change the estimate.
**Very low quality:**  very uncertain about the estimate.

^1^ Downgraded three levels for risk of bias (ROB-2 some concerns) and imprecision (zero events and wide confidence interval)

^2^ Downgraded two levels for risk of bias (ROB-2 some concerns) and imprecision (confidence interval overlaps no effect)

^3^ Downgraded two level for risk of bias (ROB-2 some concerns) and inconsistency (heterogeneity)

^4^ Downgraded three levels for risk of bias (ROB-2 some concerns), inconsistency (heterogeneity) and imprecision (confidence interval overlaps no effect)

^5^ Downgraded three levels for risk of bias (ROB-2 some concerns), inconsistency (heterogeneity) and imprecision (confidence interval overlaps no effect)

^6^ Downgraded one level for risk of bias (ROB-2 some concerns)

**Table 6: GRADE Assessment for RCT studies comparing Gasless vs Open technique**

| **GENERAL SURGERY** | | | | | | | | | | |
| --- | --- | --- | --- | --- | --- | --- | --- | --- | --- | --- |
|  |  | **Certainty assessment** | | | | **Number of patients** | | **Effect** | | **Certainty** |
| **Outcome** | **№ of studies** | **Risk of bias** | **Inconsistency** | **Indirectness** | **Imprecision** | **Gasless** | **Open** | **Relative (95% CI)** | **Absolute (95% CI)** | **GRADE** |
| Intraoperative complications | 1 | Very serious | Not serious | Very serious | Serious | 1/5 (20%) | 0/5 (0%) | **RR 3.0 (0.15 to 59.89)** | **0 fewer per 1,000** (from 0 fewer to 0 fewer) | ⨁◯◯◯ VERY LOW^1^ |
| Overall complications | 1 | Very serious | Not serious | Very serious | Serious | 1/95 (1.1%) | 1/95 (1.1%) | **RR 1.00** (0.08 to 11.93) | **0 fewer per 1,000** (from 10 fewer to 115 more) | ⨁◯◯◯ VERY LOW^1^ |
| Operative time | 1 | Very serious | Not serious | Not serious | Serious | 5 | 5 | - | **MD 10 higher** (0.64 higher to 19.36 higher) | ⨁◯◯◯ VERY LOW^1^ |
| Length of stay | 2 | Very serious | Not serious | Not serious | Serious | 145 | 145 | - | **MD 2.46 lower** (5.23 lower to 0.3 higher) | ⨁◯◯◯ VERY LOW^1^ |
| **GYNAECOLOGY** | | | | | | | | | | |
| Intraoperative complications | 2 | Serious | Serious | Not serious | Very serious | 0/90 (0%) | 0/90 (0%) | Not estimable |  | ⨁◯◯◯ VERY LOW^1^ |
| Conversion rate | 2 | Serious | Not serious | Not serious | Very serious | 0/90 (0%) | 0/90 (0%) | Not estimable |  | ⨁◯◯◯ VERY LOW^1^ |
| Overall complications | 2 | Serious | Serious | Not serious | Very serious | 0/90 (0%) | 0/90 (0%) | Not estimable |  | ⨁◯◯◯ VERY LOW^1^ |
| Operative time | 2 | Serious | Not serious | Not serious | Serious | 90 | 90 | - | **MD 18.74 lower (**29.23 lower to 8.6 lower) | ⨁◯◯◯ VERY LOW^1^ |
| Length of stay | 2 | Serious | Not serious | Not serious | Serious | 90 | 90 | - | **MD 0.2 higher** (0.04 lower to 0.44 higher) | ⨁◯◯◯ VERY LOW^1^ |

**CI:** Confidence interval; **MD:** Mean difference; **RR:** Risk ratio

GRADE Working Group grades of evidence

**High quality:** Further research is very unlikely to change our confidence in the estimate of effect.
**Moderate quality:** Further research is likely to have an important impact on our confidence in the estimate of effect and may change the estimate.
**Low quality:** Further research is very likely to have an important impact on our confidence in the estimate of effect and is likely to change the estimate.
**Very low quality:** Very uncertain about the estimate.

^1^ Downgraded three levels for risk of bias (ROB-2 some concerns) and imprecision (limited number of participants and wide confidence interval overlaps with no effect)

^2^ Downgraded three levels for risk of bias (ROB-2 some concerns), inconsistency (heterogeneity) and imprecision (confidence interval overlaps no effect)

^3^ Downgraded one level for imprecision (zero events)

^4^ Downgraded one level for inconsistency (heterogeneity)

^5^ Downgraded one level for imprecision (confidence interval overlaps no effect)

**TABLE 7: GRADE assessment for Gasless vs conventional - non-RCTs (comparative cohort studies)**

| **GENERAL SURGERY** | | | | | | | | | | |
| --- | --- | --- | --- | --- | --- | --- | --- | --- | --- | --- |
|  |  | **Certainty of evidence** | | | | **Number of patients** | | **Effect** | | **Certainty** |
| **Outcomes** | **№ of studies** | **Risk of bias** | **Inconsistency** | **Indirectness** | **Imprecision** | **Gasless** | **Conventional** | **Relative (95% CI)** | **Absolute (95% CI)** | **GRADE** |
| Conversion rate | 4 | Serious | Not serious | Not serious | Serious | 13/187 (7.0%) | 13/137 (9.5%) | **RR 0.86** (0.41 to 1.83) | **13 fewer per 1,000** (from 56 fewer to 79 more) | ⨁⨁◯◯ LOW^1,4^ |
| Overall complications | 4 | Very serious | Not serious | Not serious | Serious | 13/187 (7.0%) | 15/129 (11.6%) | **RR 0.62** (0.24 to 1.64) | **44 fewer per 1,000** (from 88 fewer to 74 more) | ⨁◯◯◯ VERY LOW^1,4^ |
| Operative time | 5 | Serious | Serious | Not serious | Serious | 207 | 148 | - | **MD 8.53 higher** (4.68 higher to 12.38 higher) | ⨁◯◯◯ VERY LOW^1,2,4^ |
| Length of stay | 7 | Serious | Not serious | Not serious | Serious | 133 | 71 | - | **MD 0.04 lower** (0.88 lower to 0.8 higher) | ⨁⨁◯◯ LOW^1,4^ |
| **GYNAECOLOGY** | | | | | | | | | | |
| Conversion rate | 7 | Serious | Not serious | Not serious | Serious | 2/247 (0.8%) | 3/322 (0.9%) | **RR 0.90** (0.15 to 5.21) | **1 fewer per 1,000** (from 8 fewer to 39 more) | ⨁⨁◯◯ LOW^1,4^ |
| Overall complications | 2 | Serious | Serious | Serious | Serious | 0/98 (0.0%) | 3/132 (2.3%) | **RR 0.23** (0.01 to 4.28) | **28 fewer per 1,000** (from 23 fewer to 75 more) | ⨁◯◯◯ VERY LOW^1-4^ |
| Operative time | 6 | Serious | Serious | Serious | Serious | 253 | 313 | - | **MD 8.16 higher** (1.87 lower to 18.19 higher) | ⨁◯◯◯ VERY LOW^1-4^ |
| Length of stay | 4 | Serious | Not serious | Not serious | Serious | 117 | 146 | - | **MD 0.1 lower** (0.02 lower to 0.22 higher) | ⨁⨁◯◯ LOW^1,4^ |

**CI:** Confidence interval; **MD:** Mean difference; **RR:** Risk ratio

GRADE Working Group grades of evidence
**High quality:** Further research is very unlikely to change our confidence in the estimate of effect.
**Moderate quality:** Further research is likely to have an important impact on our confidence in the estimate of effect and may change the estimate.
**Low quality:** Further research is very likely to have an important impact on our confidence in the estimate of effect and is likely to change the estimate.
**Very low quality:** We are very uncertain about the estimate.

^1^Risk of bias

- If you think any limitations were negligible choose no
- If you think there were serious limitations choose serious «this will downgrade the quality of evidence for this outcome by 1 level »
- If you think there were very serious limitations choose very serious «this will downgrade the quality of evidence for this outcome by 2 levels »

^2^Inconsistency

- If you think any inconsistency was negligible choose no
- If you think there was serious inconsistency choose serious
  «this will downgrade the quality of evidence for this outcome by 1 level »
- If you think there was very serious inconsistency choose very serious
  «this will downgrade the quality of evidence for this outcome by 2 levels »

^3^Indirectness

- If you think the evidence is direct choose no
- If you have serious doubts about directness choose serious
  «this will downgrade the evidence for this outcome by 1 level »
- If you have very serious doubts about directness choose very serious
  «this will downgrade the evidence for this outcome by 2 levels »

^4^Imprecision

- If you think the results were precise choose no
- If there was serious imprecision choose serious
  «this will downgrade the quality of evidence for this outcome by 1 level »
- If there was very serious imprecision choose very serious
  «this will downgrade the quality of evidence for this outcome by 2 levels »

**TABLE 8: GRADE assessment for Gasless vs open - non-RCTs (comparative cohort studies)**

| **GENERAL SURGERY** | | | | | | | | | | |
| --- | --- | --- | --- | --- | --- | --- | --- | --- | --- | --- |
|  |  | **Certainty of evidence** | | | | **Number of patients** | | **Effect** | | **Certainty** |
| **Outcomes** | **№ of studies** | **Risk of bias** | **Inconsistency** | **Indirectness** | **Imprecision** | **Gasless** | **Open** | **Relative (95% CI)** | **Absolute (95% CI)** | **GRADE** |
| Operative time | 8 | Serious | Serious | Not serious | Serious | 137 | 165 | - | **MD 3.83 higher** (22.52 lower to 30.8 higher) | ⨁◯◯◯ VERY LOW^1,2,4^ |
| Overall complications | 4 | Serious | Not serious | Not serious | Serious | 8/79 (10.1%) | 14/107 (13.1%) | **RR 0.84** (0.34 to 2.06) | **21 fewer per 1,000** (from 86 fewer to 139 more) | ⨁⨁◯◯ LOW^1,4^ |
| Length of stay | 8 | Serious | Serious | Not serious | Not serious | 152 | 178 | - | **MD 3.94 lower** (5.93 lower to 1.95 lower) | ⨁⨁◯◯ LOW^1,2^ |
| **GYNAECOLOGY** | | | | | | | | | | |
| Operative time | 3 | Serious | Serious | Not serious | Serious | 113 | 114 | - | MD 25.11 **higher** (3.34 lower to 53.55 higher) | ⨁◯◯◯ VERY LOW^1,2,4^ |
| Overall complications | 7 | Serious | Not serious | Not serious | Serious | 12/113 (10.6%) | 15/114 (13.2%) | **RR 0.82** (0.41 to 1.66) | **24 fewer per 1,000** (from 78 fewer to 87 more) | ⨁⨁◯◯ LOW^1,4^ |
| Length of stay | 3 | Serious | Serious | Not serious | Serious | 113 | 114 | - | **MD 1.75 lower** (2.64 lower to 0.86 lower) | ⨁◯◯◯ VERY LOW^1,2,4^ |

**CI:** Confidence interval; **MD:** Mean difference; **RR:** Risk ratio

GRADE Working Group grades of evidence
**High quality:** Further research is very unlikely to change our confidence in the estimate of effect.
**Moderate quality:** Further research is likely to have an important impact on our confidence in the estimate of effect and may change the estimate.
**Low quality:** Further research is very likely to have an important impact on our confidence in the estimate of effect and is likely to change the estimate.
**Very low quality:** We are very uncertain about the estimate.

^1^Risk of bias

- If you think any limitations were negligible choose no
- If you think there were serious limitations choose serious «this will downgrade the quality of evidence for this outcome by 1 level »
- If you think there were very serious limitations choose very serious «this will downgrade the quality of evidence for this outcome by 2 levels »

^2^Inconsistency

- If you think any inconsistency was negligible choose no
- If you think there was serious inconsistency choose serious
  «this will downgrade the quality of evidence for this outcome by 1 level »
- If you think there was very serious inconsistency choose very serious
  «this will downgrade the quality of evidence for this outcome by 2 levels »

^3^Indirectness

- If you think the evidence is direct choose no
- If you have serious doubts about directness choose serious
  «this will downgrade the evidence for this outcome by 1 level »
- If you have very serious doubts about directness choose very serious
  «this will downgrade the evidence for this outcome by 2 levels »

^4^Imprecision

- If you think the results were precise choose no
- If there was serious imprecision choose serious
  «this will downgrade the quality of evidence for this outcome by 1 level »
- If there was very serious imprecision choose very serious
  «this will downgrade the quality of evidence for this outcome by 2 levels »
