# Supplementary material for: Clinical effectiveness of gasless laparoscopic surgery for abdominal conditions: systematic review and meta-analysis
Source: Surg Endosc. 2021 Aug 16;35(12):6427–37. doi: 10.1007/s00464-021-08677-7 (PMC8599349; doi:10.1007/s00464-021-08677-7)
Supplement: Supplementary file 2 — Supplementary file2 (DOCX 4129 kb) [file 464_2021_8677_MOESM2_ESM.docx]

**Clinical effectiveness of gasless laparoscopic surgery for abdominal conditions: systematic review and meta-analysis**

N Aruparayil MD^1^, W Bolton MBChB^1^, A Mishra MD^2^, L Bains MD^2^, J Gnanaraj MCh^3^, R King PhD^4^, Professor T Ensor PhD^4^, N King MSc^5^, Professor D Jayne MD^1^, B Shinkins PhD^5^

^1^Leeds Institute of Medical Research at St. James’s, University of Leeds, UK

^2^Maulana Azad Medical College, Delhi, India

^3^Karunya University, Coimbatore, India

^4^Nuffield Centre for International Health and Development, Leeds Institute of Health Sciences, University of Leeds, UK

^5^Academic Unit of Health Economics, Leeds Institute of Health Sciences, University of Leeds, UK

KEYWORDS

*Gasless laparoscopy, abdominal wall lift, LMIC, low resource setting, clinical effectiveness, open surgery, general surgery, gynaecological surgery*

Address of Correspondence:

Mr Noel Aruparayil

NIHR Global Health Research Group – Surgical Technologies

Clinical Sciences Building

Level 7, Room 7.19

Leeds

LS9 7TF

[n.k.aruparayil@leeds.c.uk](mailto:n.k.aruparayil@leeds.c.uk)

+447540775214

**Figure 2: Forest plot comparing intraoperative (procedure-related) complications in studies comparing gasless versus conventional laparoscopic surgery. Risk ratios are shown with 95 per cent confidence intervals.**

**Figure 3: Subgroup analysis of conversion rate in studies comparing gasless vs conventional laparoscopic surgery. Risk ratios are shown with 95 per cent confidence intervals**

**Figure 4: Funnel plot for conversion rate comparing gasless versus conventional laparoscopy demonstrating symmetrical ‘inverted funnel shape’ taking into consideration random effects model to incorporate heterogeneity in the meta-analysis and demonstrating low publication bias.**

**Figure 5: Estimate of overall complications in studies comparing gasless vs conventional laparoscopic surgery. Risk ratios are shown with 95 per cent confidence intervals**

**Figure 6: Estimate of overall complications in studies comparing gasless vs open surgery. An Inverse Variance random‐effects model was used for meta‐analysis. Risk ratios are shown with 95 per cent confidence intervals.**

**Figure 7: Subgroup analysis of operative time in studies comparing gasless vs conventional laparoscopic surgery. Mean differences are shown with 95 per cent confidence intervals**

**Figure 8: Subgroup analysis of studies comparing operative time in gasless vs open surgery. An Inverse Variance random‐effects model was used for meta‐analysis. Mean differences are shown with 95 per cent confidence intervals**

**Figure 9: Subgroup analysis of studies comparing Length of Hospital Stay (LoS) in gasless vs conventional laparoscopic surgery. An Inverse Variance random‐effects model was used for meta‐analysis. Mean differences are shown with 95 per cent confidence intervals**

**Figure 10: Subgroup analysis of studies comparing the length of stay in gasless vs open surgery. An Inverse Variance random‐effects model was used for meta‐analysis. Mean differences are shown with 95 per cent confidence intervals**

**Figure 11: LMIC Subgroup analysis of operative time in studies comparing gasless vs conventional laparoscopic surgery. Mean differences are shown with 95 per cent confidence intervals.**

**Figure 12: LMIC subgroup analysis of conversion rate in studies comparing gasless vs conventional laparoscopic surgery. Risk ratios are shown with 95 per cent confidence intervals**

**Figure 13: LMIC subgroup analysis of overall complications in studies comparing gasless vs conventional laparoscopic surgery. Risk ratios are shown with 95 per cent confidence intervals**
