# Supplementary material for: Clinical effectiveness of gasless laparoscopic surgery for abdominal conditions: systematic review and meta-analysis
Source: Surg Endosc. 2021 Aug 16;35(12):6427–37. doi: 10.1007/s00464-021-08677-7 (PMC8599349; doi:10.1007/s00464-021-08677-7)
Supplement: Supplementary file 3 — Supplementary file3 (DOCX 16 kb) [file 464_2021_8677_MOESM3_ESM.docx]

**Clinical effectiveness of gasless laparoscopic surgery for abdominal conditions: systematic review and meta-analysis**

N Aruparayil MD^1^, W Bolton MBChB^1^, A Mishra MD^2^, L Bains MD^2^, J Gnanaraj MCh^3^, R King PhD^4^, Professor T Ensor PhD^4^, N King MSc^5^, Professor D Jayne MD^1^, B Shinkins PhD^5^

^1^Leeds Institute of Medical Research at St. James’s, University of Leeds, UK

^2^Maulana Azad Medical College, Delhi, India

^3^Karunya University, Coimbatore, India

^4^Nuffield Centre for International Health and Development, Leeds Institute of Health Sciences, University of Leeds, UK

^5^Academic Unit of Health Economics, Leeds Institute of Health Sciences, University of Leeds, UK

KEYWORDS

*Gasless laparoscopy, abdominal wall lift, LMIC, low resource setting, clinical effectiveness, open surgery, general surgery, gynaecological surgery*

Address of Correspondence:

Mr Noel Aruparayil

NIHR Global Health Research Group – Surgical Technologies

Clinical Sciences Building

Level 7, Room 7.19

Leeds

LS9 7TF

[n.k.aruparayil@leeds.c.uk](mailto:n.k.aruparayil@leeds.c.uk)

+447540775214

**SEARCH STRATEGIES**

S1

Search strategies:

Database: Ovid MEDLINE(R) ALL <1946 to January 22, 2021>

Search Strategy:

--------------------------------------------------------------------------------

1 (laparoscop* or coelioscop* or celioscop* or peritoneoscop*).ab,ti. (132560)

2 exp Laparoscopy/ (100384)

3 Minimally Invasive Surgical Procedures/ (26298)

4 (Minimal* adj3 (invasiv* or access* or surg*)).tw. (79339)

5 1 or 2 or 3 or 4 (219204)

6 (gasless* or isobaric or non-insufflat* or noninsufflat* or insufflation-less*).ab,ti. (6515)

7 (laparolift* or laparofan or laparotensor or Kirschner wires or variolift or abdolift or kents clamp* or hoffman's trocar or lifting arm mizuho or AWL Hashimoto or AWL aesculap).ab,ti. (1676)

8 ((abdomen or abdominal) adj4 lift*).tw. (314)

9 ((lift* or non-CO2 or CO2-free) adj4 laparoscop*).tw. (104)

10 6 or 7 or 8 or 9 (8434)

11 5 and 10 (713)

12 (breast or prostat* or urolog* or nephr* or thoracic or endocrine or thyroid).ab,ti. (1316063)

13 11 not 12 (612)

***************************

Database: Embase Classic+Embase <1947 to 2021 January 21>

Search Strategy:

--------------------------------------------------------------------------------

1 (laparoscop* or coelioscop* or celioscop* or peritoneoscop*).ab,ti. (214724)

2 exp Laparoscopy/ (168106)

3 Minimally Invasive Surgery/ (43953)

4 (Minimal* adj3 (invasiv* or access* or surg*)).tw. (119578)

5 1 or 2 or 3 or 4 (351973)

6 (gasless* or isobaric or non-insufflat* or noninsufflat* or insufflation-less*).ab,ti. (7589)

7 (laparolift* or laparofan or laparotensor or Kirschner wires or variolift or abdolift or kents clamp* or hoffman's trocar or lifting arm mizuho or AWL Hashimoto or AWL aesculap).ab,ti. (2041)

8 ((abdomen or abdominal) adj4 lift*).tw. (469)

9 ((lift* or non-CO2 or CO2-free) adj4 laparoscop*).tw. (126)

10 6 or 7 or 8 or 9 (10009)

11 5 and 10 (922)

12 (breast or prostat* or urolog* or nephr* or thoracic or endocrine or thyroid).ab,ti. (1950301)

13 11 not 12 (777)

14 limit 13 to conference abstracts (115)

15 13 not 14 (662)
